# *IDH1*-mutant vaccine in newly diagnosed astrocytoma: final analysis of the multicenter, single-arm, open-label, first-in-human phase 1 NOA16 trial

**DOI:** 10.1038/s43018-026-01199-y

**Published:** 2026-07-01

**Authors:** Lukas Bunse, Katharina Lindner, Antje Wick, Angelika Freitag, Lisa-Marie Lanz, Dominic Edelmann, Abigail Suwala, Michael O. Breckwoldt, Inga Harting, Felix Sahm, Richard F. Schlenk, Anita Schmitt, Oliver Schnell, Jörg Hense, Martin Misch, Dietmar Krex, Monika Denk, Juliane Walz, Joachim P. Steinbach, Andreas von Deimling, Michael Schmitt, Theresa Bunse, Ghazaleh Tabatabai, Martin Bendszus, Isabel Poschke, Wolfgang Wick, Michael Platten

**Affiliations:** 1 DKTK (German Cancer Consortium) Clinical Cooperation Unit (CCU) Neuroimmunology and Brain Tumor Immunology, German Cancer Research Center (DKFZ), Heidelberg, Germany; 2 Department of Neurology, Medical Faculty Mannheim, MCTN, University of Heidelberg, Mannheim, Germany; 3 German Cancer Consortium (DKTK), DKFZ, Core Center, Heidelberg, Germany; 4 Hertie Network of Excellence in Clinical Neuroscience, Tübingen, Germany; 5 DKFZ-Hector Cancer Institute at University Medical Center Mannheim, Mannheim, Germany; 6 Cluster of Excellence SynthImmune (EXC 3018/1) “Engineering Immune function with Synthetic Biology”, University of Heidelberg, Mannheim, Germany; 7 Immune Monitoring Unit, National Center for Tumor Diseases (NCT), Heidelberg, Germany; 8 Neurology Clinic and European Center for Neurooncology (EZN), Heidelberg University Hospital, University of Heidelberg, Heidelberg, Germany; 9 NCT Trial Management and Services, NCT, and German Cancer Research Center (DKFZ), Heidelberg, Germany; 10 Department of Neuropathology, Heidelberg University Hospital, University of Heidelberg, Heidelberg, Germany; 11 Department of Neuroradiology, Heidelberg University Hospital, University of Heidelberg, Heidelberg, Germany; 12 DKTK CCU Neuropathology, DKFZ, Heidelberg, Germany; 13 Department of Medical Oncology, Heidelberg University Hospital, University of Heidelberg, Heidelberg, Germany; 14 Department of Internal Medicine V, Heidelberg University Hospital, University of Heidelberg, Heidelberg, Germany; 15 Neurosurgery Clinic, University Hospital Erlangen, Erlangen, Germany; 16 Department of Medical Oncology, West German Cancer Center, University Hospital Essen, University of Duisburg-Essen, Essen, Germany; 17 Department of Neurosurgery, Charité Medical Center, University of Berlin, Berlin, Germany; 18 Technische Universität Dresden, Faculty of Medicine and University Hospital Carl Gustav Carus, Department of Neurosurgery, Dresden, Germany; 19 Cluster of Excellence iFIT (EXC2180) ‘Image-Guided and Functionally Instructed Tumor Therapies’, University of Tübingen, Tübingen, Germany; 20 Department of Peptide-based Immunotherapy, Institute of Immunology, University and University Hospital Tübingen, Tübingen, Germany; 21 German Cancer Consortium (DKTK) and German Cancer Research Center (DKFZ), partner site Tübingen, Tübingen, Germany; 22 Dr. Senckenberg Institute of Neurooncology, Frankfurt, Germany; 23 Department of Neurology and Neuro-Oncology, University Hospital Tübingen, Hertie Institute for Clinical Brain Research, Eberhard Karls University Tübingen, Tübingen, Germany; 24 Center for Neuro-Oncology, CCC Tübingen-Stuttgart in CCC-SW, Tübingen, Germany; 25 DKTK CCU Neurooncology, DKFZ, Heidelberg, Germany; 26 Helmholtz Institute for Translational Oncology (HI-TRON) Mainz, German Cancer Research Center, Mainz, Germany

**Keywords:** CNS cancer, CNS cancer, Tumour immunology, Peptide vaccines, Cancer

## Abstract

The clonal glioma driver mutation *IDH1*^R132H^ gives rise to a major histocompatibility class II-restricted neoepitope. A multicenter, first-in-human phase 1 trial met its prespecified primary endpoints by demonstrating safety and immunogenicity of an IDH1-R132H peptide vaccine (IDH1-vac) integrated into standard of care in 33 participants with newly diagnosed grade III and IV (World Health Organization classification 2007) IDH1-R132H^+^ astrocytomas (NOA16). Here we report on the clinical and immunological long-term follow-up of this trial as secondary and translational endpoints. The 8-year progression-free and overall survival (OS) rates were 0.42 (confidence interval (CI): 0.24–0.59) and 0.66 (CI: 0.46–0.79), respectively. For participants with grade IV astrocytoma, median OS was 106.1 months (CI: 39.6–not estimable (NE)), comparing favorably to the published median OS in this population ranging from 31.6–56.4 months. Within the responder group, sustained antibody responses to IDH1-R132H were associated with a favorable long-term clinical course. IDH1-vac-induced T cell responses were detected in the inflamed brain lesion of an IDH1-vac-associated pseudoprogression, whereas no IDH1-vac-induced T cells were found in participants with early progressive disease. The favorable long-term outcome of the NOA16 cohort supports investigating IDH1-vac in persons with newly diagnosed grade 3 and 4 (World Health Organization classification 2021) *IDH*-mutant astrocytomas in a randomized phase 2 trial (ClinicalTrials.gov identifier: NCT02454634).

## Abstract (web summary)

In the multicenter, single-arm, open-label, first-in-human phase 1 NOA16 trial, Platten and colleagues report the long-term clinical and immunological follow-up outcomes of a vaccine targeting mutant IDH1 in persons with astrocytoma.

## Main

Mutations in the genes of isocitrate dehydrogenase (IDH) 1 and 2 are present in nearly all astrocytomas and oligodendrogliomas. Most frequently, these mutations result in the neomorphic protein IDH1-R132H, where arginine is substituted to histidine at position 132 in IDH1 (ref. ^1^). Grading of *IDH*-mutant gliomas has been changed over time. Here, Roman numerals refer to the World Health Organization (WHO) classification from 2007, which was used for NOA16, and Arabic numerals refer to the current central nervous system (CNS) WHO classification from 2021. In persons with CNS WHO grade 2 astrocytoma with no other treatment than surgery, vorasidenib, an oral brain-penetrant inhibitor of mutant IDH1/2, recently showed improved progression-free survival (PFS) and delayed time to the next intervention in a randomized double-blinded phase 3 clinical trial^2^. As very first molecular therapy in this population, vorasidenib has received US Food and Drug Administration and European Medicines Agency approval. With the concept of reprogramming and normalizing *IDH*-mutant glioma cells, IDH small-molecule inhibitors^3,4^ are thought to be particularly efficacious at early disease stages. However, malignant transformation is still inevitable and CNS WHO grade 3–4 IDH-mutant tumors, despite adjuvant radiochemotherapy, remain incurable. A 20-mer peptide vaccine against IDH1-R132H (IDH1-vac) has been developed in major histocompatibility complex (MHC) humanized mice^5^ and was assessed in the first-in-human open-label phase 1 clinical trial NOA16. NOA16 demonstrated the safety and immunogenicity of IDH1-vac in 32 participants with WHO grade III and IV astrocytoma (WHO 2007) independent of human leukocyte antigen allelotypes and in combination with standard of care (SOC) radiotherapy and/or chemotherapy^6^. Here we report on the 8-year clinical and immunological long-term follow-up of the NOA16 cohort, correlate immune responses to long-term clinical outcomes and provide a first rationale of an IDH1-vac maintenance therapy.

## Results

### Long-term clinical outcomes in participants following treatment with IDH1-vac

**Fig. 1:**
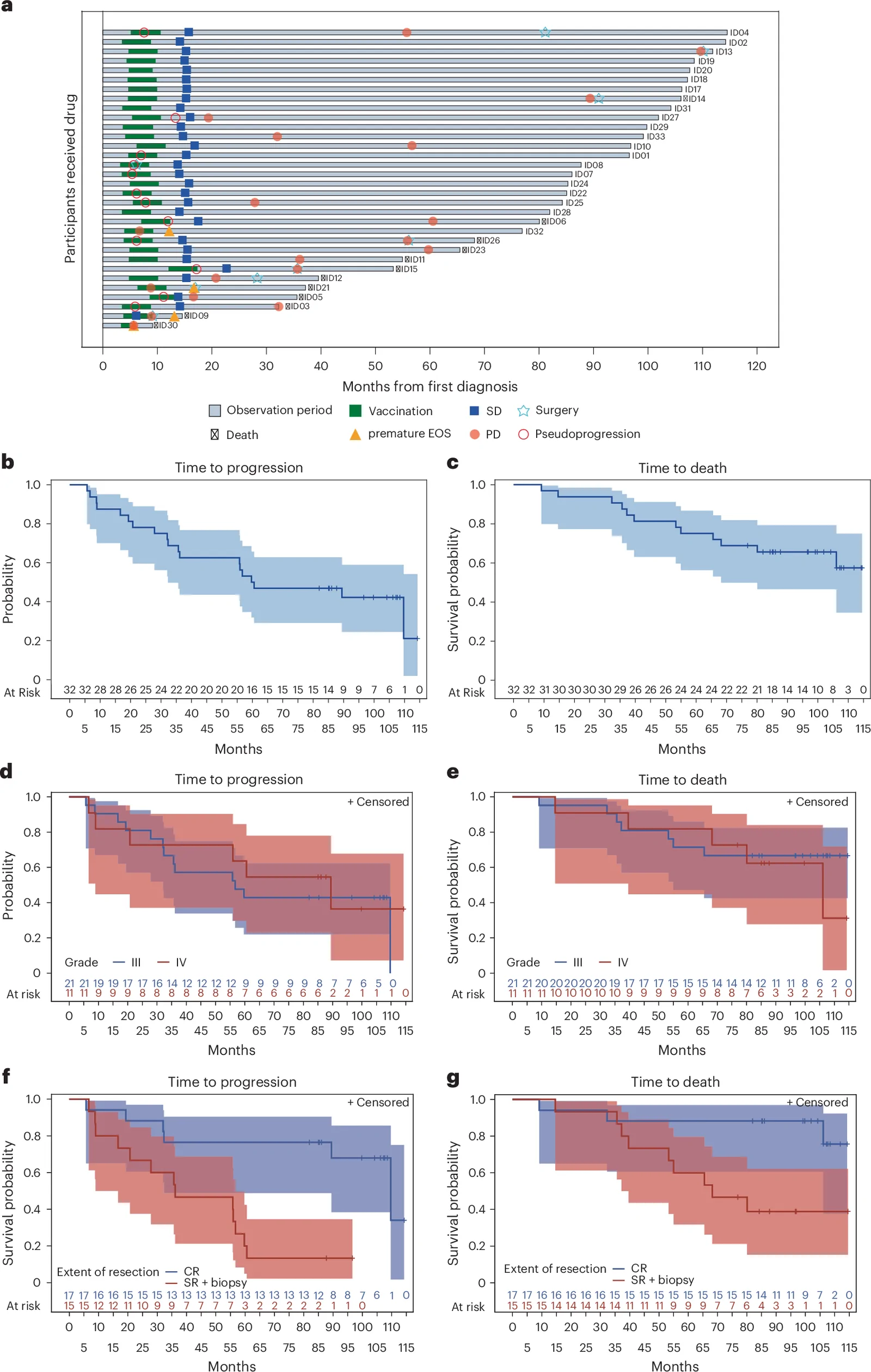
Long-term clinical course of participants receiving IDH1-vac. **a**, Swimmer plot depicting disease progression (PD) and interventions for each participant in the SDS (*n* = 32 participants). For every participant, disease status was assessed in 3–6-month intervals. Disease status was once assessed also at EOT as visualized. **b**,**c**, PFS (left) and OS (right) estimates with number of participants at risk shown for all participants of the SDS. **d**,**e**, PFS (left) and OS (right) estimates with number of participants at risk shown for all participants of the SDS according to WHO grade (WHO classification 2007). **f**,**g**, PFS (left) and OS (right) estimates with number of participants at risk shown according to extent of resection. SR, subtotal resection. The 95% CI is displayed in **b**–**g**.

In NOA16^6^, 33 participants with newly diagnosed astrocytoma, IDH-mutant, grade III and IV, were enrolled and 32 participants (safety population) received IDH1-vac in combination with SOC radiotherapy and/or chemotherapy (radiotherapy and temozolomide (TMZ) (*n* = 23, 71.9%), TMZ alone (*n* = 3, 9.4%) or radiotherapy alone (*n* = 6, 18.8%)) (Fig. 1a). A total of 21 (65.6%) participants had WHO grade III astrocytoma, *IDH*-mutant and 11 (34.4%) participants had grade IV astrocytoma (according to WHO classification 2007). A total of 17 participants (53.1%) had undergone complete resection (CR) of the tumor, 12 (37.5%) had undergone subtotal resection and three (9.4%) had undergone a biopsy only. Median follow-up was 99.8 months (confidence interval (CI): 86.1–107.2). The 8-year PFS and overall survival (OS) rates of participants in the safety dataset (SDS; *n* = 32) were 0.42 (CI: 0.24–0.59) and 0.66 (CI: 0.46–0.79), respectively (Fig. 1b,c). The median PFS was 60.1 months (CI: 32.3–not estimable (NE)) and after the median OS was not reached after 8 years.

The 8-year PFS and OS rates in the grade III population were 0.43 (CI: 0.22–0.62) and 0.68 (CI: 0.43–0.84), respectively (Fig. 1d,e). The 8-year PFS and OS rates in participants with grade IV astrocytomas were 0.36 (CI: 0.07–0.68) and 0.62 (CI: 0.28–0.84), respectively. The median PFS was 56.7 months (CI: 32.0–NE) for participants with grade III and 89.4 months (CI: 8.9–NE) for participants with grade IV astrocytomas. The median OS for participants with grade III astrocytoma was still not reached after 8 years. For participants with grade IV astrocytoma, the median OS was 106.1 months (CI: 39.6–NE).

In participants with grade III and IV astrocytoma, the *IDH*-mutant and CR (n = 17) 8-year PFS and OS rates were 0.68 (CI: 0.38–0.86) and 0.88 (CI: 0.60–0.97), respectively (Fig. 1f,g). For participants with CR, the median PFS was 109.7 months (CI: 32.3–NE), whereas the median OS was not reached (cutoff: February 2025).

**Extended Data Fig. 1:**
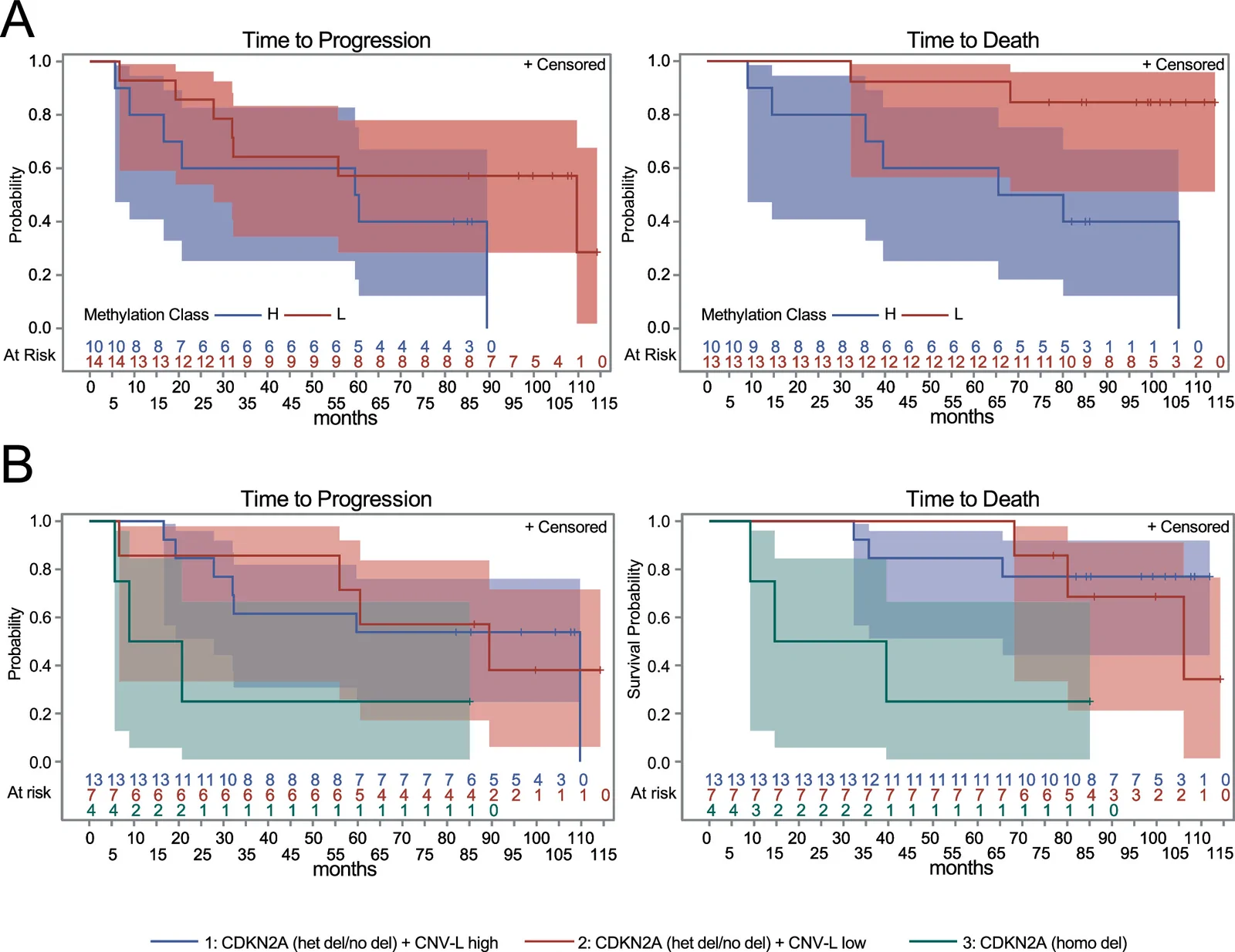
Probabilities of progression and death in the NOA16 population according to methylation class and CNV load-based grouping. **A)** Progression-free (left) and overall (right) survival estimates with number of patients at risk are shown according to methylation class (molecular set, n = 24). **B)** Progression-free (left) and overall (right) survival estimates with number of patients at risk are shown according to CDKN2A/B status and CNV-load (CNV-L). n(CDKN2A/B het del and no del + CNV-L high) = 4; n(CDKN2A/B het del and no del + CNV-L low) = 16; n(CDKN2A/B homo) = 4. 95% Confidence Interval is displayed in A-B. Source data

Methylation class-based grading was determined retrospectively for 24 of 32 participants (75.0%). A low-grade methylation class was found in 14 astrocytomas (58.3%) and, in the remaining ten (41.7%) astrocytomas, a high-grade methylation class was found. The 8-year PFS and OS rates in participants with a low-grade methylation class (*n* = 14) were 0.57 (CI: 0.28–0.78) and 0.86 (CI: 0.54–0.96), respectively. The median PFS and OS in the high-grade methylation class subpopulation were 60.1 months (CI: 5.7–NE) and 72.8 months (CI: 9.1–NE), respectively (Extended Data Fig. 1). While this outcome compares favorably to published cohorts stratified by methylation^7–9^, the small sample size, differences in cutoff values and molecular and clinical confounders limit the relevance of this comparison.

### Association of immune response to clinical outcome

**Fig. 2:**
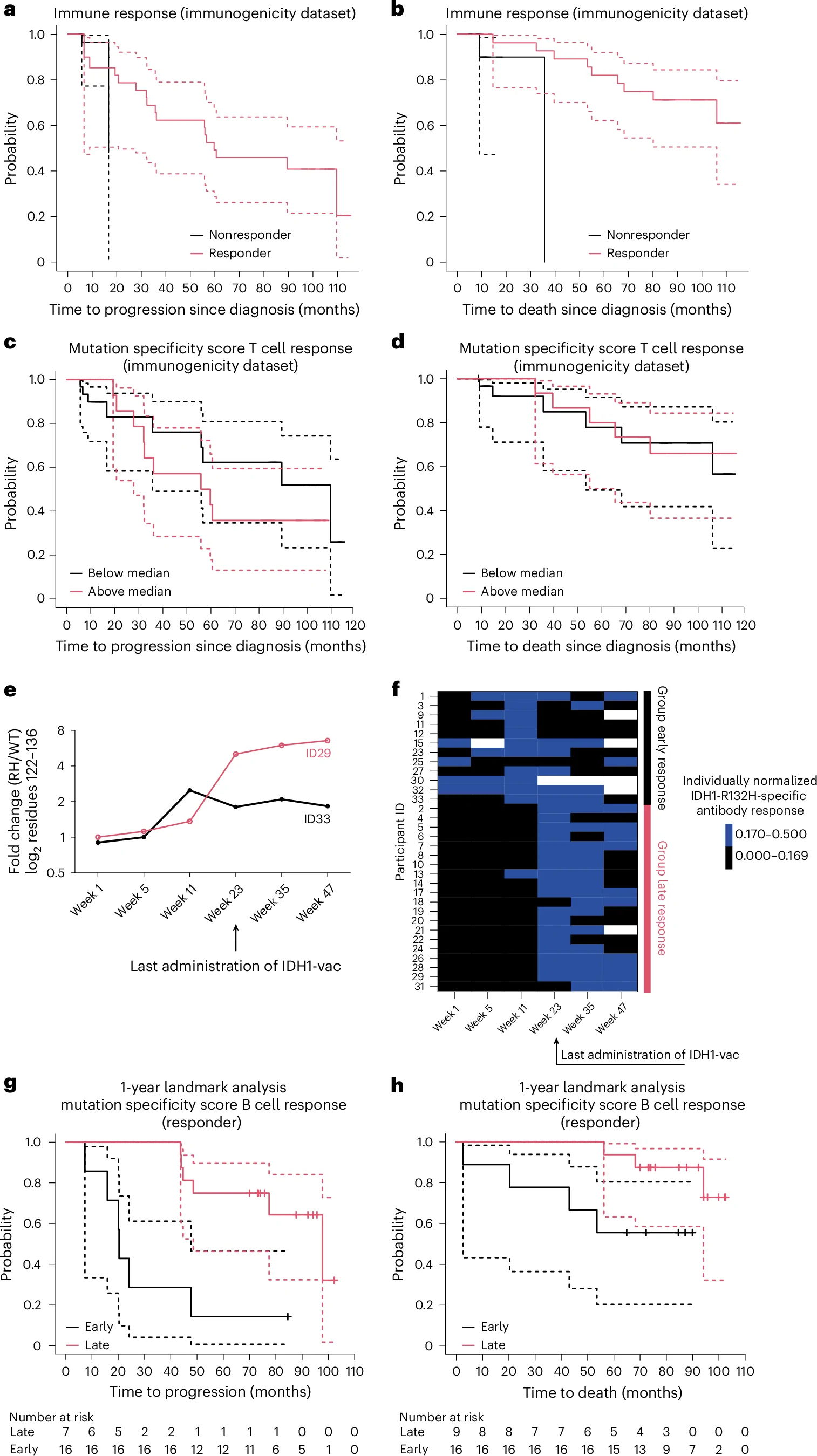
T and B cell response as time-dependent variables in the NOA16 immunogenicity population. **a**,**b**, Simon and Makuch plot of PFS and OS probabilities according to the time-dependent covariate IDH1-vac-induced immune response (*n* = 30 participants). The *x* axes show the time since first diagnosis. The number of participants at risk is indicated. **c**,**d**, Simon and Makuch plot of PFS and OS probabilities according to the time-dependent covariate T-MSS above and below median (IDS, *n* = 30). The *x* axes show the time since first diagnosis. The number of participants at risk is indicated. **e**, Exemplary longitudinal peptide-coated serum ELISA of participants ID29 and ID33. Week 1, baseline before vaccination; week 47, EOS. Relative values are shown. Each time point was assessed in technical triplicates. **f**, Participant individually normalized dynamics of mutation-specific (RH/WT) antibody titers over the duration of the repetitive vaccinations. Week 1, baseline before vaccination; week 23, time point of last vaccination; week 47, EOS. Early versus late mutation-specific B cell responders were compared. The two sampling time points with the highest mutation-specific B cell response before versus at or after last vaccination time point were used to define groups. **g**,**h**, Simon and Makuch plot of PFS and OS probabilities according to the time-dependent covariate early versus late mutation-specific B cell responders (IDS, *n* = 30). The *x* axes show the time since first diagnosis. The number of participants at risk is indicated.

**Extended Data Fig. 2:**
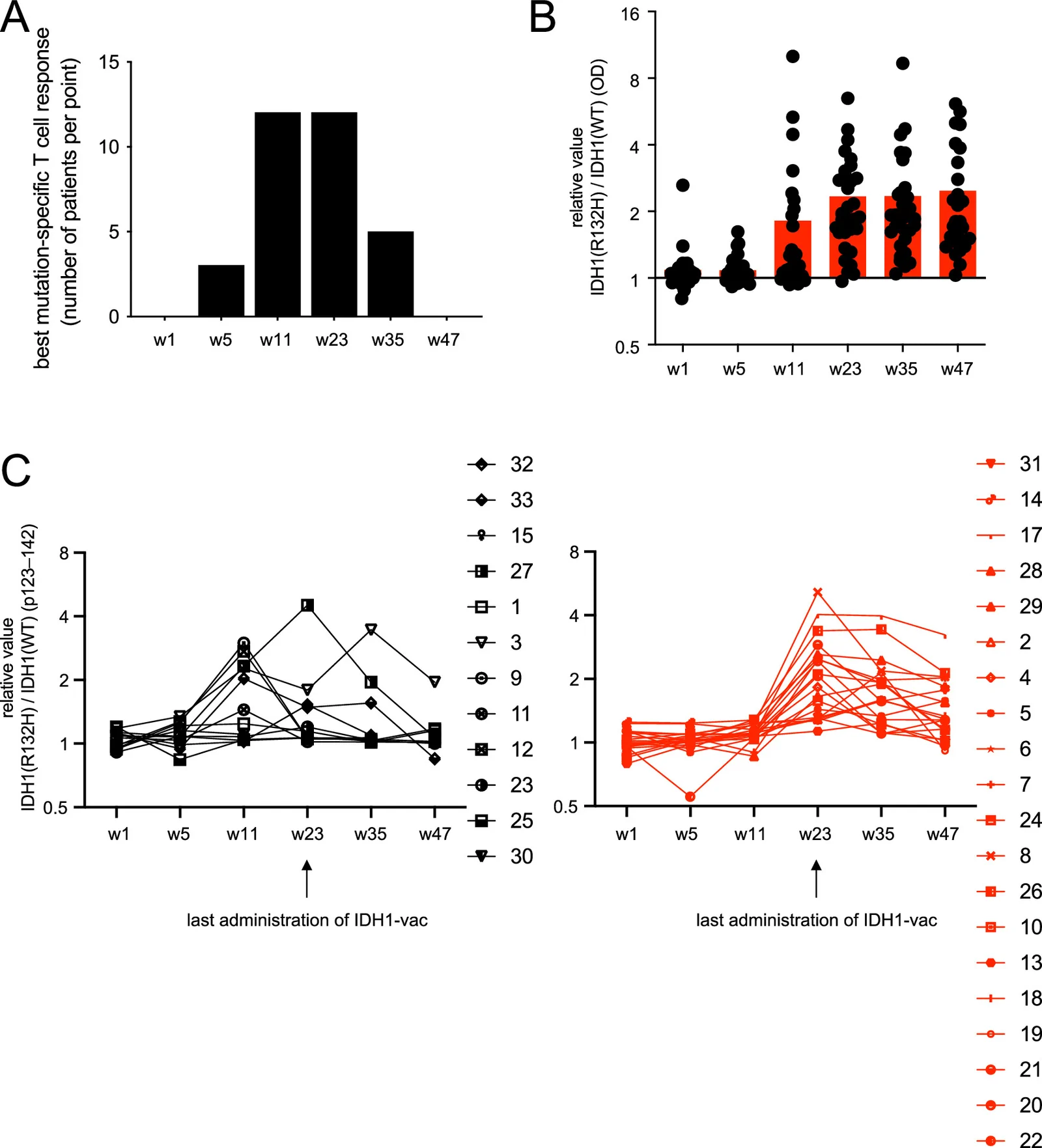
Cumulative on-trial dynamics of T and B cell responses. **A)** Frequency of best IDH1(R132H)-reactive T cell response at different time points until EOS (w47) assessed by ELISpot assay. Safety dataset is shown (n = 32). **B)** IDH1(R132H)-specific B cell response (relative value) at different time points until EOS (w47) assessed by ELISA. Safety dataset is shown (n = 32). **C)** Longitudinal B cell response stratified according to best responses pre and with or post last vaccination. Time point of last vaccination indicated by an arrow.

**Extended Data Fig. 3:**
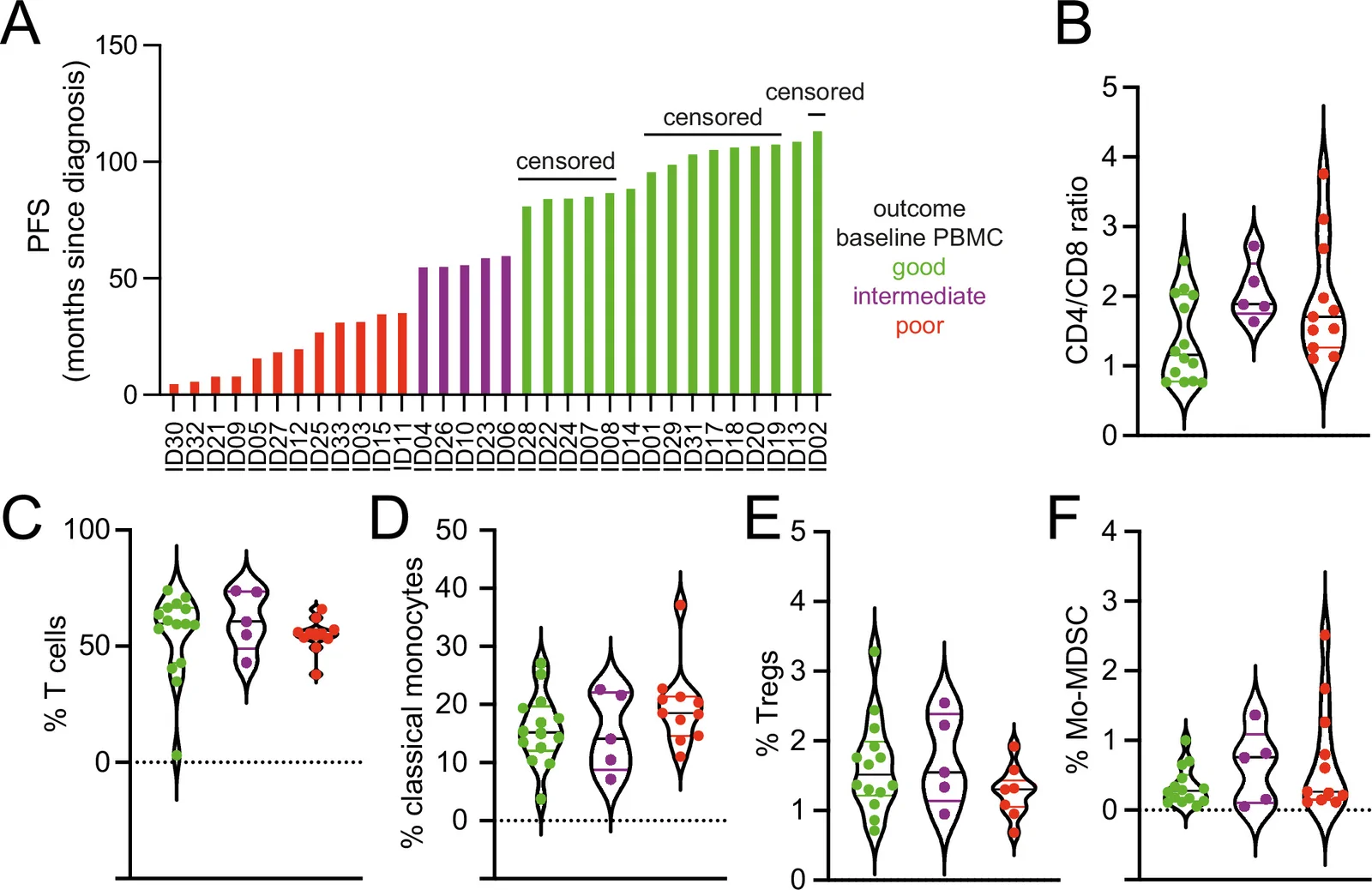
Correlation of clinical outcome and baseline proportion of peripheral immune cells. **A)** According to PFS, patients can be grouped according to good, intermediate, and poor outcome (data cut-off March 2025). **B-F)** Baseline proportion of CD4/CD8 T cell ratio, T cells, monocytes, regulatory T cells, and monocytic myeloid-derived suppressor cells (Mo-MDSCs) in PBMC according to clinical outcome assessed by flow cytometry.

In the initial trial report, exploratory analyses suggested biological activity of IDH1-vac^6^. First, there was a strong positive correlation of intratumoral IDH1-R132H peptide presentation in the tumor tissue with the magnitude and sustainability of specific peripheral T cell responses. Second, compared to a molecularly controlled cohort of 60 participants receiving SOC, 37.5% (12/32) of NOA16 participants experienced pseudoprogression (PsPD) versus 16.7% (10/60) in the control cohort. Third, in one of the participants with PsPD, vaccine-induced IDH1-R132H-reactive T cells were retrieved from a post-treatment resected inflammatory lesion, linking the presence of IDH1-vac-induced T cells to PsPD^10^. With the 8-year PFS and OS rates at hand, we aimed to investigate whether adaptive immune responses to IDH1(R132H) assessed during the trial were associated with long-term clinical outcomes, even though the IDH1-vac was only given during the trial for 6 months. Two participants without immune response showed progressive disease (PD) within 2 years and died within 3 years of first diagnosis (Fig. 2a,b). In contrast, a landmark analysis revealed that, in participants with an IDH1-vac-induced immune response in the first year, the OS probability 8 years later was still over 50%. As IDH1-vac treatment elicits both T cell (responders: 87.5% (28/32)) and B cell (responders: 93.75% (30/32)) responses and as both responses were elicited in most participants (81.3%, 26/32), we next aimed to correlate clinical long-term outcomes to the quality and dynamic of T or B cell responses. Overall, T cell response peaked at week 23 and started to decline at week 35, which is the first time point after vaccination—12 weeks after the last vaccination (Extended Data Fig. 2). At year 8 after IDH1-vac treatment, PFS and OS rates were similar in participants who showed a T cell mutation specificity score (T-MSS) above versus below median (Fig. 2c,d). T-MSS^6^ was previously defined considering both the on-trial duration and the mutation specificity of the T cell response during IDH1-vac treatment. In contrast to the T cell responses, IDH1-R132H-specific antibody responses assessed by ELISA were generally more sustained (Extended Data Fig. 2). Interestingly, IDH1-R132H-specific antibody responses varied between participants, peaking before the last vaccination in some participants, while others developed sustained IDH1-R132H-specific responses upon the last vaccination or later (Fig. 2e,f). Comparing participant outcome between the two groups, the PFS and OS were improved in the participants that developed their best IDH1-R132H-specific antibody response upon the last vaccination time point or later, likely profiting from repetitive vaccinations (Fig. 2g,h and Extended Data Fig. 2). To exclude confounding factors such as the proportion of immunosuppressive peripheral cell populations at baseline, we correlated long-term clinical outcomes to peripheral immune cell phenotypes but did not find explainable differences (Extended Data Fig. 3). In summary, sustained immune response during the trial, particularly a humoral IDH1-R132H-specific response, correlated positively with a favorable long-term clinical course in the NOA16 population.

### TCR repertoires and specificities in progression and PsPD

**Fig. 3:**
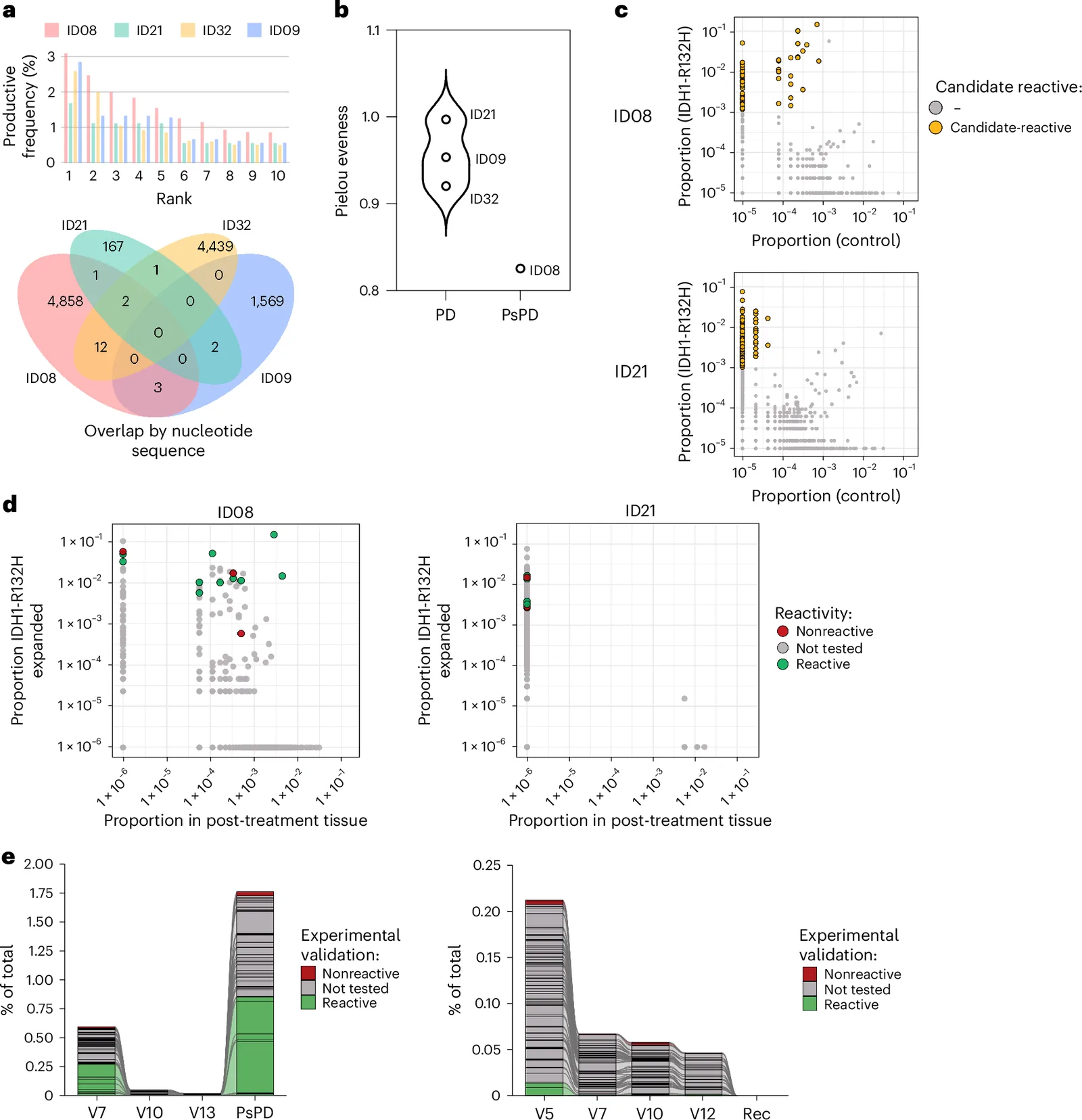
TCR repertoires and specificities in progressive and pseudoprogressive lesions. **a**, TCR β deep sequencing of post-treatment tissues (top ten TCR clonotypes (top) and Venn diagram (bottom) from participants ID08 (PsPD), ID09, ID21 and ID32 (all PD). **b**, Pielou evenness (Shannon index divided by the log of the unique number of clonotypes) of PsPD (ID08) versus PD (ID09, ID21 and ID32). **c**, Comparative peptide-based deep TCR β sequencing of PBMCs from participants ID08 and ID21 following IDH1(R132H) peptide-based expansion and no peptide control. TCRs labeled in orange were above the predefined cutoff (no peptide: <10^−3^; IDH1-R132H: >10^−3^).**d**, Proportions of functionally validated IDH1-R132H-reactive T cells in tissues (ID08, PsPD; ID21, PD) and in IDH1-R132H peptide-expanded PBMC-derived T cells. **e**, Clonal evolution of IDH1-R132H-reactive T cells during repetitive vaccinations and in post-treatment tissues as indicated. Clones are defined on the amino acid level. V5, V7, V10 and V13 represents participant visits per the study protocol^6^. V5 is the first postvaccine immunomonitoring time point.

**Extended Data Fig. 4:**
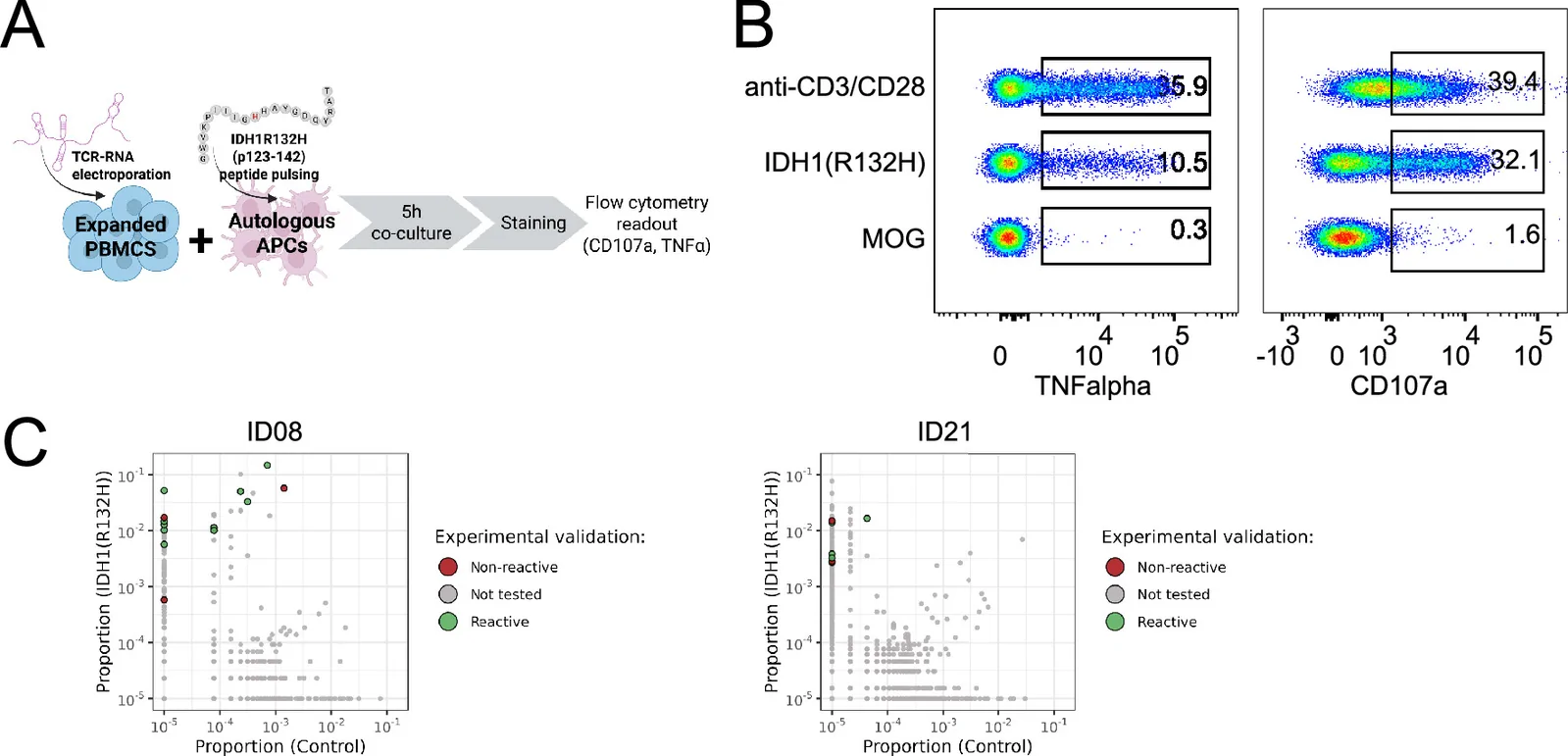
Functional validation of IDH1(R132)-reactive T cell receptors. **A)** Workflow of functional TCR validation from preselected TCRs as in C. TCRs are cloned and in vitro transcribed into RNA and electroporated into patient autologous PBMC. Subsequently, TCR-engineered PBMCs are co-cultured with cognate peptide-loaded antigen. **B)** Flow cytometric quantification of CD107a and TNFα expression is used to assess target-specific activation. **C)** Visualization of functionally tested TCRs from C and E with paired alpha/beta TCR information.

We previously showed that transcriptionally defined inflammatory IDH1-R132H-reactive T cells are present in PsPD. To investigate whether PD is associated with the transient nature of IDH1-vac-induced T cell responses or dysfunction of intratumoral IDH1-R132H-reactive T cells, we performed T cell receptor (TCR) bulk sequencing of three histologically confirmed recurrent diseases (ID09, ID21 and ID32) and compared these to the molecularly characterized PsPD from participant ID08. Productive frequencies of the top ten TCRs in each sample were comparable with no relevant TCR sequence overlap (Fig. 3a). Interestingly, the TCR repertoire evenness of PsPD was markedly lower in comparison to PD, suggesting clonal expansion in PsPD (Fig. 3b). As we did not have access to freshly isolated tumor-infiltrating lymphocytes (TILs) for full TIL α/β TCR reconstruction, we aimed to identify participant-individual peripheral TCR β sequences reactive to IDH1-R132H to assess their abundance within the post-treatment PD versus PsPD tissues. To this end, we performed peptide-based expansion of peripheral T cells^11^ from participants ID08 and ID21 and subjected these to TCR β deep and droplet-based single-cell TCR sequencing (Fig. 3c). Expanded TCR clonotypes were retrieved, cloned and electroporated by in vitro transcribed RNA into expanded syngeneic peripheral T cells (Extended Data Fig. 4). TCRs that mediated CD107a expression and TNF production after coculture of electroporated T cells with IDH1-R132H peptide-loaded cells were subsequently defined as IDH1-R132H-reactive TCRs (Extended Data Fig. 4). When mapping these participant-individual IDH1-R132H-reactive TCRs back to the corresponding tissues of participants ID08 (PsPD) and ID21 (PD), IDH1-R132H-reactive T cells were exclusively found in PsPD but not in the histologically confirmed PD (Fig. 3d). Interestingly, in ID08, IDH1-R132H-reactive T cells were found in the peripheral blood after vaccination start, declined over time and were subsequently found to be clonally expanded in PsPD lesion. In contrast, in ID21, IDH1-R132H-reactive T cells were found in the peripheral blood after vaccination start, continuously declined in the peripheral blood and were subsequently absent in the recurrent tumor (Fig. 3e). Overall, our data suggest that, in some persons, infiltration of vaccine-induced T cells into the tumor are associated with PsPD but not PD.

### Long-term dynamics of T and B cell responses

**Fig. 4:**
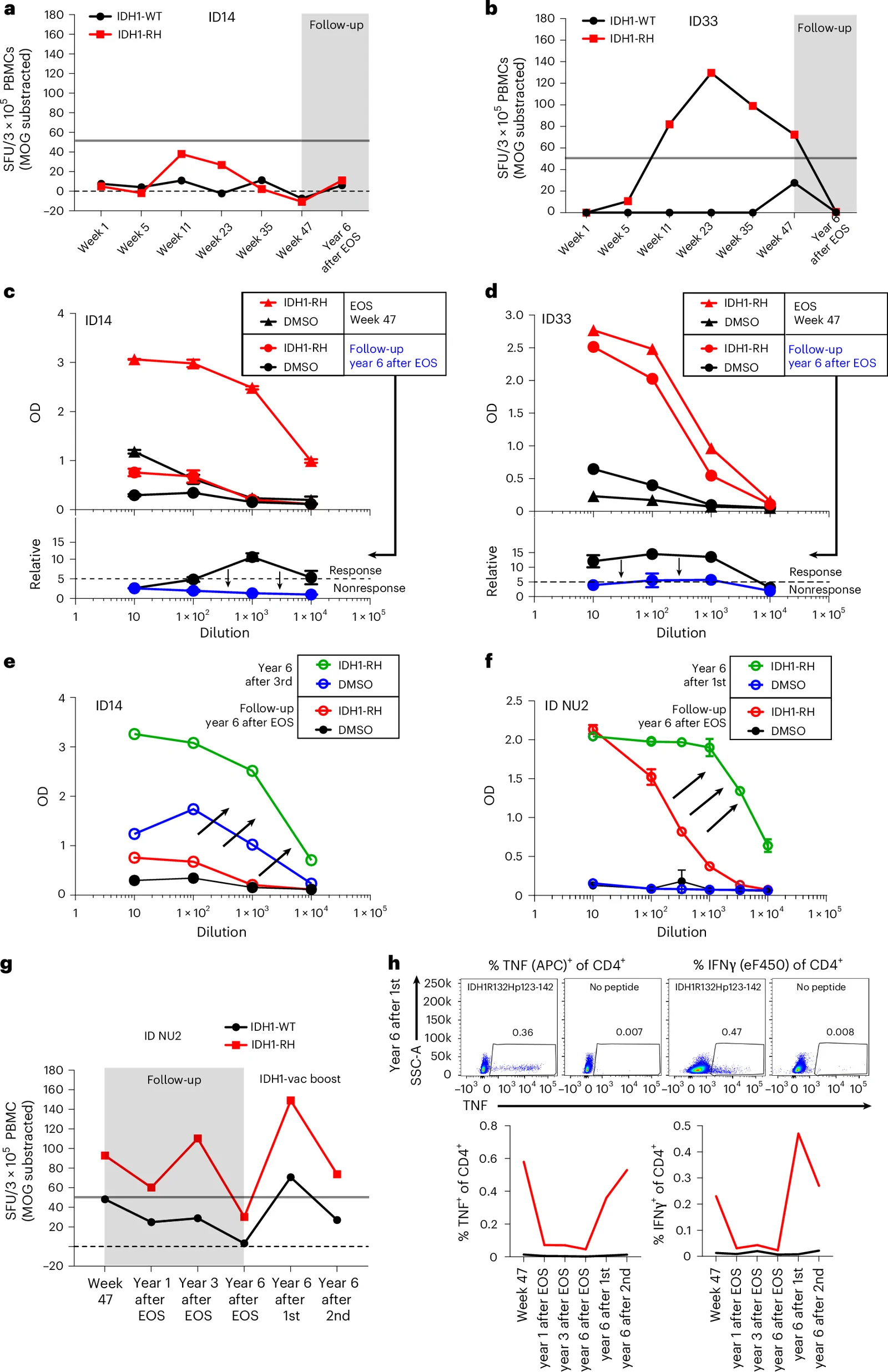
Booster IDH1-vac treatment. **a**,**b**, ELISpot assays of PBMC from participants ID14 and ID33 restimulated with IDH1-R132H and IDH1-WT peptide as indicated. SFUs following MOG stimulation (negative control) were subtracted. Week 47, EOS. **c**,**d**, IDH1-R132H-coated and vehicle (DMSO)-coated serum ELISA of participants ID14 and ID33 at different dilutions as indicated. Top, optical density (OD); bottom, relative value (IDH1-R132H/vehicle (DMSO)) at indicated dilutions. Arrows illustrate weakened IDH1-R132H-reactive B cell response at follow-up (cutoff: fivefold induction over vehicle). **e**, IDH1-R132H-coated and vehicle (DMSO)-coated serum ELISA of participant ID14 at different dilutions as indicated. Arrows illustrate an enhanced IDH1-R132H-reactive B cell response following IDH1-vac boost treatment. **f**, IDH1-R132H-coated and vehicle (DMSO)-coated serum ELISA of participant ID NU2 at different dilutions as indicated. Arrows illustrate the enhanced IDH1-R132H-reactive B cell response following IDH1-vac boost treatment. **g**, ELISpot assay of PBMC from participant ID NU2 restimulated with IDH1-R132H and IDH1-WT peptide as indicated. SFUs following MOG stimulation (negative control) were subtracted. Week 47, EOS. IDH1-vac boost treatment started 6 years after EOS. **g**, Longitudinal intracellular flow cytometry of PBMC T helper cells following in vitro restimulation with the IDH1-R132H peptide at indicated time points. Top, exemplary dot plots; bottom, quantification.

**Extended Data Fig. 5:**
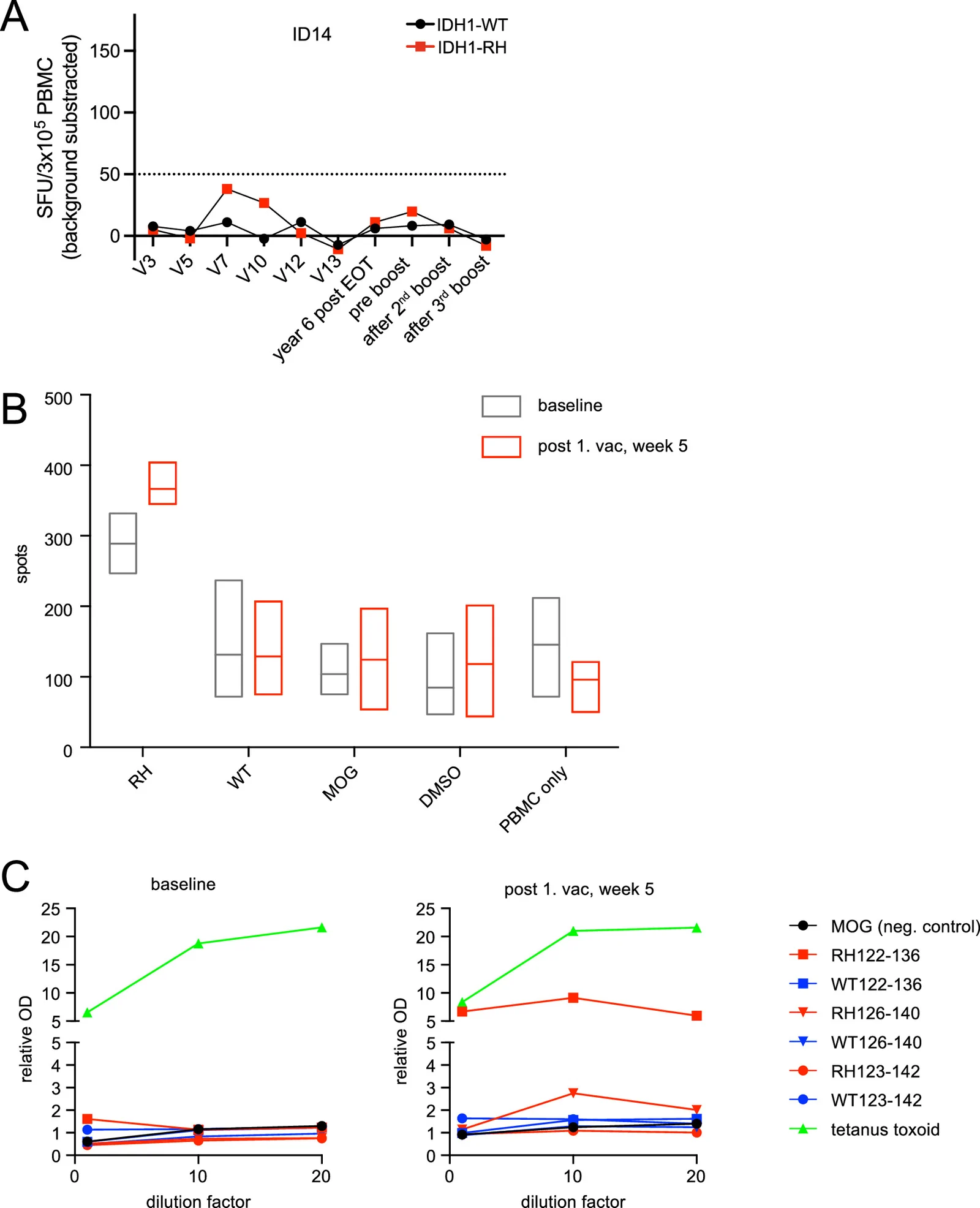
Baseline and post vaccination T and B cell responses in selected patients. **A)** IDH1(R132H)-specific T cell response during IDH1-vac booster in non-responding patient. ELISpot assays of PBMC from patient ID14 restimulated with IDH1(R132H), IDH1(WT) as indicated. SFU resulting from (MOG) stimulation (negative control/background) are subtracted. **B)** Spontaneous T cell response against IDH1(R132H) in patient ID NU2. ELISpot assays of PBMC from patient ID NU2 restimulated with IDH1(R132H), IDH1(WT), and MOG peptides and DMSO (vehicle control) as indicated. Baseline and first post-IDH1-vac time point visualized as floating bars (min to max and line at mean). **C)** IDH1(R132H) (15-mer and 20-mer peptide)-, IDH1(WT) (15-mer and 20-mer peptide)-, DMSO (vehicle)-, MOG-, tetanus toxoid (positive control)-coated serum ELISA of patient ID NU2 at different dilutions as indicated. Baseline and first post-IDH1-vac time point visualized.

As the NOA16 immune monitoring dataset suggested an association of sustained IDH1-vac-induced immune responses with favorable clinical outcome, we investigated the impact of a maintenance vaccination therapy. Peripheral T and B cell responses from two assessable on-trial immune responder participants, ID14 (only B cell response) and ID33 (T and B cell response), were monitored for 6 years after end of treatment (EOT). At that time, peripheral IDH1-R132H-specific T cell responses were still not detected in ID14 (Fig. 4a,b), while an IDH1-R132H-reactive B cell response, although reduced to end of study (EOS) below the positivity cutoff, was still detectable (Fig. 4c,d). To investigate whether reduced IDH1-R132H-reactive B cell responses can be boosted in principle, we administered three additional vaccines to participant ID14. Indeed, IDH1-R132H-specific antibody responses were boosted (titer: 1:10,000 versus 1:100 at follow-up), as measured by ELISA (Fig. 4e), without any adverse events according to Common Terminology Criteria for Adverse Events (CTCAE) version 5.0 until further progression. After the IDH1-vac booster, however, the participant remained a T cell nonresponder (Extended Data Fig. 5). Next, we asked whether an IDH1-vac booster is also safe in persons displaying B and T cell responses. Therefore, we treated a female participant with astrocytoma, WHO grade 3 (IDNU2), in analogy to the NOA16 treatment protocol. Interestingly, at baseline, this participant harbored an IDH1-R132H-specific endogenous T but not B cell response (Extended Data Fig. 5). Then, 6 years following initial diagnosis and SOC plus IDH1-vac, a booster IDH1-vac treatment was initiated at stable disease. Indeed, both B and T cell IDH1-R132H-specific responses as measured by ELISA (Fig. 3f), enzyme-linked immunospot (ELISpot) assay (Fig. 4g**)** and intracellular flow cytometry (Fig. 4h) were boosted without any adverse events. IDNU2 currently remains stable 9 years after initial diagnosis. In summary, long-term immune monitoring in three participants suggests that, years after completion of eight repetitive doses of IDH1-vac, peripheral T cell responses against IDH1-R132H only persisted in a participant with spontaneous immune responses against IDH1-R132H and that booster IDH1-vac treatments could result in a continuous peripheral source of IDH1-R132H-specific T cells and/or antibodies.

## Discussion

The long-term outcome data of NOA16 demonstrate that integration of a long-peptide vaccine targeting a clonal driver mutation into SOC of persons with newly diagnosed astrocytoma is associated with meaningful peripheral and intratumoral immune responses and a favorable clinical outcome, specifically in persons with CR (Fig. 1f,g), global immune response (Fig. 2a,b) and sustained antibody responses (Fig. 2g,h). A positive association of IDH1-vac-associated immune response with favorable clinical course in persons with IDH-mutant glioma was recently reported by others^12^ in an uncontrolled named participant use program. While data from this program generally support the concept of IDH1-R132H vaccination for persons with glioma, our study provides long-term follow-up data from a prospective multicenter national phase 1 trial with IDH1-vac as the sole experimental treatment and functional validation of vaccine-induced IDH1-R132H-reactive TCRs. Moreover, we carefully rationalized vaccination regimes with thorough reverse translation immunomonitoring.

In line with the updated WHO classifications in 2016 and 2021 (ref. ^13^), molecular grading applying *CDKN2A*/*B* deletion status was prognostic in a subset of the NOA16 cohort (Fig. 1 and Extended Data Fig. 1). Of note, this was not the case for histological grading according to WHO 2007. To better delineate the predictive value of these molecular markers for the treatment of IDH1-vac an investigation in larger cohorts is required. In line with previous reports^14^, extent of resection is an obvious and strong clinical prognostic factor, demonstrating the important aspect of macroscopically CR (whenever feasible). As *IDH*-mutant gliomas are characterized by an immunosuppressive microenvironment, particularly associated with the neomorphic enzymatic activity of mutant IDH and its oncometabolite (*R*)-2-hydroxyglutarate^15–22^, a macroscopic CR may facilitate the infiltration and local effector function of IDH1-vac-induced immune cells.

**Extended Data Fig. 6:**
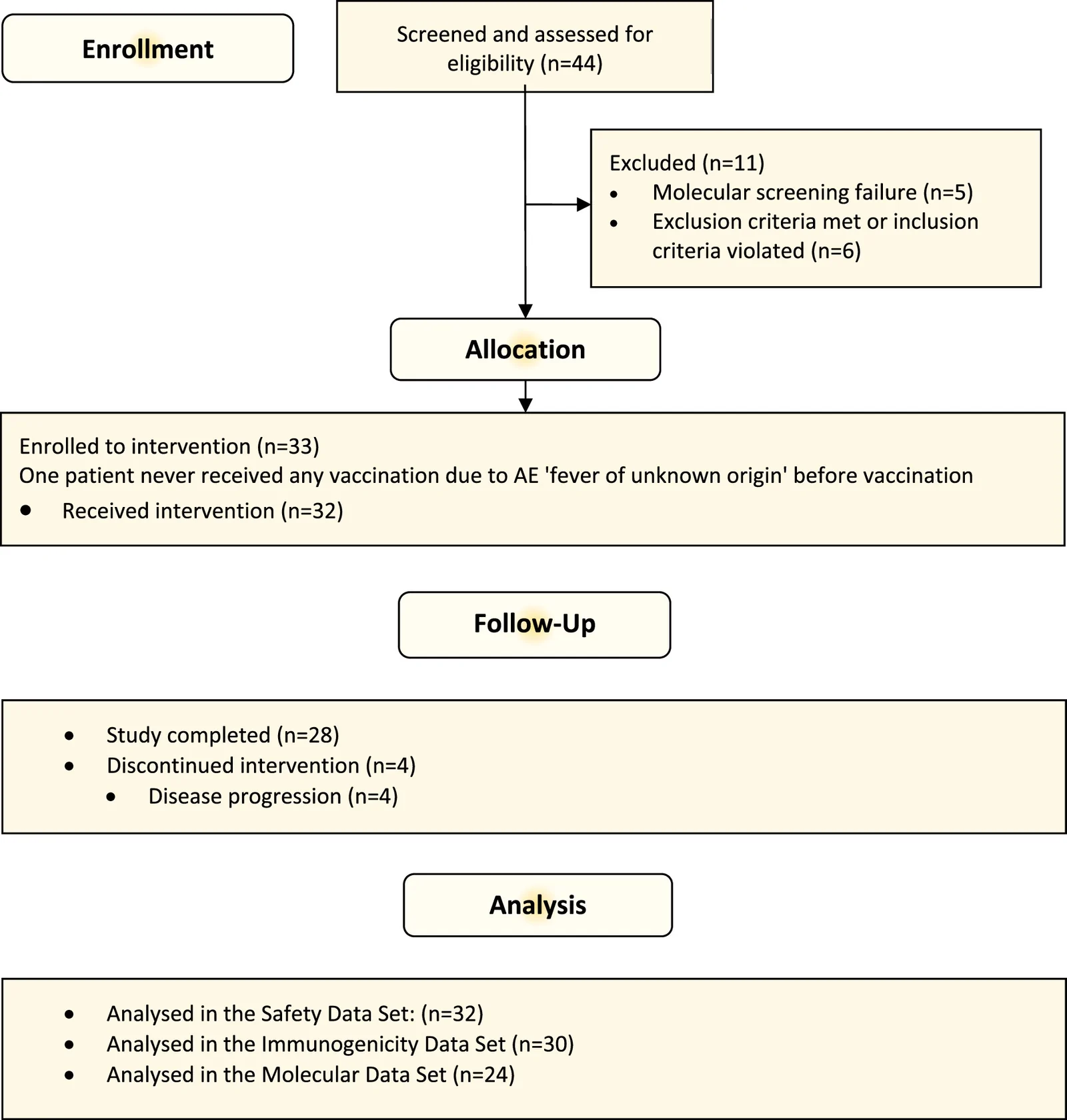
Patient disposition CONSORT flow diagram of the NOA16 trial.

The comparison of long-term clinical outcomes of the NOA16 population (Extended Data Fig. 6) to external, multiinstitutional and historical cohorts is challenging as grading of *IDH*-mutant gliomas has been substantially revised from the WHO classification 2007, which was the basis for this trial, to more recent revisions in 2016 and 2021, resulting in critical limitations. Nevertheless, the median survival rate of participants enrolled in the CATNON (EORTC 26053-22054) trial^23^ with low-grade and high-grade methylation class IDH1-R132H^+^ gliomas was 7.0 and 5.3 years^24^, respectively. In NOA16, the median OS has still not been reached after 8 years, although 41.7% of the assessable participants were classified as having high-grade methylation class tumors (Fig. 2b). For participants with grade IV *IDH*-mutant astrocytoma enrolled in NOA16, the median OS was 106.1 months, whereas the median OS ranged from 31.6 to 56.4 months in other studies^7,25–28^. We acknowledge that the selection bias and enrichment of known prognostic confounders, such as the extent of resection, in this single-arm study, as well asthe differences in grading in these studies according to the respective WHO classifications applied, present critical limitations for such comparisons. Although NOA16 was not designed to demonstrate efficacy, it demonstrates that the immune response to the IDH1-vac is associated with a favorable clinical outcome and provides a strong rationale for trial design and endpoint definitions of a phase 2 clinical study in participants with newly diagnosed CNS WHO grade 3 and 4 astrocytoma. We identified vaccine-induced IDH1-R132H-reactive clonally expanded T cells in the tissue of a participant with PsPD but not in a participant with PD, suggesting that these vaccine-induced T cells drive PsPD. Limited availability of tissue in this study precluded the analyses of pretreatment tumor tissue and further PsPD tissue samples. This question, however, will be addressed in the NOA21 window-of-opportunity trial^29^.

When assessing the quality of IDH1-vac-induced T cell responses on a participant individual level over 47 weeks (until EOS), there was no positive correlation of a long-term favorable clinical course and the quality of T cell response. This may be accounted for by three potential (nonexclusive) reasons: (1) the IDH1-vac-induced peripheral T cell response is not prognostic per se; (2) the IDH1-vac treatment restricted to only months but not years is too short; or (3) the window of peripheral multimodal immunomonitoring (47 weeks) is insufficient to study long-lasting memory responses. In addition, while T cell responses have been observed across all class II HLA allelotypes and paralogs, differences in affinities and avidities of peptide may constitute a relevant confounder. Moreover, while no differences in T cell responses were observed in the few persons who did not receive TMZ chemotherapy compared to those receiving radiochemotherapy^6^, we cannot exclude that TMZ may negatively impact sustained T cell responses. NOA21 will assess the biological and clinical efficacy of the vaccine in persons with recurrent *IDH1*^R132H^-mutant glioma without concomitant radiotherapy and/or chemotherapy^29^. The observations from this final analysis strongly suggest implementing an IDH1-vac boosting concept and longer immune monitoring beyond termination of the primary treatment phase in a future clinical trial. Interestingly, we found that participants whose mutation-specific antibody response peaked upon the last vaccination or later had remarkably favorable clinical courses. This observation is particularly exciting, as objective preclinical response was dependent on CD19^+^ B cells also in MHC humanized mice^5^. It is tempting to speculate that antibodies specific to MHC-bound tumor antigens are per se therapeutic. Alternatively, antibody titers may represent a surrogate of the number IDH1-vac-activated and, therefore, antigen-presenting B cells^30^. While the mechanistic underpinning of this observation needs to be investigated further, a recent study targeting the neoantigen histone H3K27M in diffuse midline gliomas provided first evidence of vaccine-induced intrathecal B cells reactive to a vaccination antigen^31,32^.

Conceptually, IDH1-vac elicits peripheral immune responses across a plethora of MHC class II allelotypes, corroborating an off-the-shelf concept for persons with CNS WHO grade 3–4 astrocytoma and potentially CNS WHO grade 3 oligodendroglioma. In addition, as a precision immunological intervention, IDH1-vac can be synergistically applied in combination with checkpoint inhibitors^29^ or small-molecule IDH inhibition in the future^21,33^. Targeting a highly expressed, clonal, shared driver mutation with a vaccine as a backbone offers notable advantages over personalized vaccine strategies targeting private neoepitopes, which are often subclonal and lowly expressed^34,35^. To substantiate the clinical activity of IDH1-vac in persons with newly diagnosed CNS WHO grade 3 and 4 astrocytoma, a sham-vaccine controlled double-blind clinical trial is required. Here, our data, although only obtained from two participants, suggest that a booster treatment regime may longer maintain IDH1-R132H-reactive T/B cell responses.

## Methods

### Study design

Sample size estimation was primarily based on the accuracy requirements for the primary endpoint immune response (responder rate) to the IDH1 peptide vaccine. Sample size was adjusted for nonevaluable participants. According to the estimation that 70% of participants evaluable for immunogenicity testing will be evaluable for all time points^36^, with 21 participants sufficient for immunogenicity testing with all time points, 30 evaluable participants needed to be enrolled. Because of the expected dropout rate of 20% (because of progression or other reasons), the plan was for 39 participants to be recruited. Among all 32 participants treated (SDS), two participants were excluded from immunogenicity testing because they were not evaluable as not enough time points were eligible for immunogenicity testing (immunogenicity dataset). A participant was predefined to be evaluable if, upon study completion and up until visit 7, they received at least four vaccinations and all blood samples were collected for immunogenicity testing or received six vaccinations and the baseline plus two other blood samples were collected for immunogenicity testing. Molecular testing was conducted retrospectively. For *n* = 24 participants, sufficient material was available for additional molecular testing. Findings concerning participant outcomes and immunogenicity at defined time points and all related analyses using participant blood samples or derivatives thereof cannot be reproduced because of sample limitations. TCR testing was reproduced at least three times with similar outcome. This was a phase 1 study, whereby all participants received IDH1 vaccination (IDH1-vac). In addition, they received SOC treatment before enrollment as decided by the local investigator and the participant. Three types of SOC treatment resulted in three treatment groups, all receiving the exact same trial-related intervention. This study was a single-arm, open-label trial, whereby neither participants nor clinical nor immunogenicity investigators were blinded concerning IDH1 vaccination (trial-related intervention). With respect to SOC treatment groups, immunogenicity investigators were blinded. All primary endpoint analyses were conducted in a blinded fashion. Exploratory analyses for example immunological phenotyping were performed nonblinded with respect to immune response detectable in the sample, because samples for these analyses were selected on the basis of the immune response. Inclusion in the trial was not restricted with regard to ethnicity or socially relevant groupings. The population was 62.5% male and 37.5% female; the mean age was 40.4 ± 8.95 years. Age, gender and peripheral immune cell counts were descriptively assessed. Participants were recruited at eight trial centers in Germany on the basis of molecular and clinical inclusion criteria: presence of a histologically confirmed IDH1-R132H^+^ glioma (with or without measurable residual tumor after resection or biopsy) with absence of chromosomal 1p/19q codeletion and loss of nuclear *ATRX* expression in the tumor tissue (subgroup of molecular astrocytoma without positive prognostic factors). In addition, inclusion criteria were as follows: participants receiving SOC treatment (RT + TMZ, TMZ alone or RT alone) before enrollment; at least 18 years old; women of child-bearing potential (WOCBP) needed to provide a negative pregnancy test within 72 h before the start of IDH1 vaccination; WOCBP and their partners had to use a birth control method (failure rate below 1% per year). Exclusion criteria were as follows: concomitant treatment with dexamethasone (or equivalent) at >2 mg per day, Karnofsky performance status < 70, PD (including PsPD) or recurrent disease after SOC treatment or experimental treatment of the tumor; grade 2 or higher CTCAE (version 4.0) laboratory values for hematology, liver or renal function. A complete list of exclusion criteria is provided in the Supplementary Data. For TCRB deep sequencing and single-cell RNA/TCR-seq, peripheral blood mononuclear cell (PBMC) and tissue samples, respectively, were selected on the basis of availability. In flow cytometry, cells were allocated to different stainings (full stain panels and staining controls) randomly.

Participants agreeing to trial methods were normally well informed and motivated to comply with study procedures, which may have influenced the results in the way of better interpretability. More information is available in our previous trial publications.

### Trial approval and ethics

The study was approved by the national regulatory authority (Paul-Ehrlich Institut) and the institutional review board (Ethik-Kommission) at each study site: Ethik-Kommission der Medizinischen Fakultät Heidelberg (Heidelberg), Ethik-Kommission Albert-Ludwigs-Universität Freiburg (Freiburg), Ethik-Kommission des Landes Berlin (Berlin), Ethik-Kommission der Medizinischen Fakultät der Universität Duisburg-Essen (Essen), Ethik-Kommission der Medizinischen Fakultät ‘Carl Gustav Carus’ (Dresden), Ethik-Kommission des Fachbereichs Medizin der Goethe-Universität Frankfurt am Main (Frankfurt), Ethik-Kommission der Medizinischen Fakultät der Ludwig-Maximilians-Universität München (Munich), Ethik-Kommission an der Medizinischen Fakultät der Eberhard-Karls-Universität and Universitätsklinikum Tübingen (Tübingen). The study was conducted in accordance with the Good Clinical Practice guidelines of the International Conference on Harmonization. All participants provided written signed informed consent. We complied with all relevant ethical regulations.

### IDH1 vaccination

IDH1-vac consisted of 300 μg of an IDH1-R132H 20-mer peptide (residues 123–142) manufactured by the Good Manufacturing Practice (GMP) facility of the University of Tübingen and emulsified in Montanide (ISA50) as described previously^5^ by the GMP core facility at the University Hospital Heidelberg a maximum of 1 day in advance or distributed as mixing kit. It was administered subcutaneously in combination with topical imiquimod (5%; Aldara). Quality controls for content, sterility and absence of endotoxin were performed for each emulsion at Labor LS.

### IFNγ ELISpot of PBMCs

ELISpot white-bottom multiscreen high-throughput sequencing plates (MSIPS4W10, Millipore) were coated with anti-human IFNγ (1-D1K, Mabtech) and blocked with X-Vivo-20 (Lonza) containing 2% human albumin. PBMCs were thawed, rested overnight in X-Vivo20 medium, seeded at 4 × 10^5^ cells per well and stimulated with 2 μg of peptides per well in a 100-μl volume. PBMCs were stimulated with IDH1-R132H (residues 123–142), wild-type IDH1 (IDH1-WT; residues 123–142) or myelin oligodendrocyte glycoprotein (MOG; residues 35–55) at equal concentrations, using peptide diluent aqua ad iniectabilia (Braun) with 10% DMSO (vehicle) at equal volume as negative controls and 1 μg of staphylococcal enterotoxin B (Sigma-Aldrich) per well and 0.05 μg of CMV with 0.05 μg of AdV per well (both in a 100-μl volume) as positive controls. After 40 h, IFNγ-producing cells were detected with biotinylated anti-human IFNγ antibodies (7-B6-1), streptavidin–ALP (both Mabtech) and ALP color development buffer (Bio-Rad) and quantified using an ImmunoSpot Analyzer (Cellular Technology). Quality control was performed and reviewed by a second person. For categorization of T cell responses, T cell responses were defined as a count of 50 spot-forming units (SFUs) above background (MOG-stimulated).

### IDH1 IgG ELISA

ELISA polysorp plates (Nunc) were coated with human IDH1-R132H and IDH1-WT (residues 122–136 and 123–142) for IgG detection or with negative control MOG (p35–55) (10 μg per well in PBS). Wells were washed with PBS 0.05% Tween-20 and blocked with 3% FBS in PBS 0.05% Tween-20. The positive control for participant serum was tetanus toxoid (Millipore) with EBNA-1 (RayBiotech) (each 0.5 ng per well). Sera from participants and healthy controls were obtained from serum tubes by centrifugation. Participant serum was used at the following dilutions: 1:10, 1:100, 1:333, 1:1,000 and 1:3,333. Healthy control serum was used undiluted. Mouse anti-IDH1-R132H (1:1,000; H09, Dianova) was used as peptide coating control. Horseradish peroxidase (HRP)-conjugated secondary antibodies were sheep anti-mouse IgG-HRP (1:5,000; Amersham) and goat anti-human IgG-Fc-HRP (1:10,000; Bethyl Laboratories). The substrate was tetramethylbenzidine (eBioscience) and the reaction was stopped with 1 M H2SO4. Optical density was measured at 450 nm.

### TCRB deep sequencing

Genomic DNA was isolated from participant EDTA blood using the DNeasy blood and tissue kit (Qiagen). TCRB deep sequencing was performed to detect rearranged TCRβ gene sequences using the hsTCRB kit (Adaptive Biotechnologies) according to the manufacturer’s protocol. The prepared library was sequenced on an Illumina MiSeq by the Genomics and Proteomics Core Facility, German Cancer Research Center (DKFZ). Data processing (demultiplexing, trimming and gene mapping) was performed using the Adaptive Biotechnologies proprietary platform.

### IDH1-R132H-reactive TCR identification and testing

To select TCRs for reactivity assessment, a peptide-specific T cell expansion assay was performed as described before^32^ and top expanded clones were selected for TCR reactivity assessment, as described previously^37^.

Briefly, CD4^+^-enriched expanded T cells from the blood of nonautologous healthy donors were thawed and rested overnight in X-Vivo15 supplemented with 2% AB serum. Before electroporation, a 48-well plate was filled with 1 ml of prewarmed medium (TexMACS supplemented with 2% AB serum) per well and placed in a 37 °C CO_2_ incubator. Cells were washed once using TexMACS medium, cell numbers were determined using trypan blue and the required number of cells was spun down (100*g*, 10 min, room temperature). Per electroporation condition, 2 × 10^6^ cells were resuspended in 20 µl of supplemented buffer P3 solution (supplemented according to the manufacturer’s protocol; Lonza, V4XP-3032) and transferred into one well of a 16-well Nucleocuvette strip containing 750 ng of TCR-encoding RNA per well. Cells were transfected with the EO-115 nucleofection program of the Lonza 4D Nucleofector and left at room temperature for 10 min for recovery, before 180 µl of prewarmed medium from the previously prepared 48-well plate was added per well of the Nucleocuvette strip. Next, 200 µl of cell suspension was resuspended once and carefully transferred back into the corresponding wells of the 48-well plate. The plate was subsequently transferred into a 37 °C CO2 incubator.

After 18–24 h, cells were harvested in tubes containing Benzonase (final concentration of 50 IU per ml) to prevent cell clumping, spun down and resuspended in X-Vivo15 supplemented with 2% HSA (human albumin 20%; Behring, P100245582). Cell numbers were determined using trypan blue and cell concentrations were set to 1.5 × 10^6^ cells per ml. After confirming mTCRβ expression through flow cytometry (viability dye (AF700, eBioscience), CD4 (clone SK3 Leu3a, BV786, BD), CD8a (clone RPA-T8, PerCP-Cy5.5, BD) and mTCRβ (clone H57-597, PE, BioLegend)), 100 µl of cells were plated in the appropriate wells of a 96-well U-bottom plate.

To test the reactivity of each transduced TCR, cocultures were performed in a 1:10 target-to-effector ratio with autologous dendritic cells that were pulsed overnight with 5–10 M of peptide MOG (negative control; MEVGWYRSPFSRVVHLYRNGK), IDH1-WT (GWVKPIIIGRHAYGDQYRAT) or IDH1-R132H (GWVKPIIIGHHAYGDQYRAT)). Anti-CD3/CD28 T cell TransAct beads (1.5 µl per well; Miltenyi, 130-111-160) served as positive controls for each tested TCR. Cocultures were set up in a total volume of 200 µl per well in X-Vivo15 supplemented with 2% HSA.

Next, 5 µl of CD107a (clone H4A3, APC-H7, BD) was added to each well, cultures were mixed by pipetting up and down and the plate was spun for 1 min at 20*g* to ensure immediate contact between expanded PBMCs and antigen-presenting cells. Cells were transferred into a 37 °C CO_2_ incubator. After 1 h, 10 µl of 1:44 prediluted GolgiStop (BD, 554724) and 10 µl of 1:44 prediluted GolgiPlug (BD, 555029) were added per well, cultures were resuspended by pipetting up and down and the plate was spun for 1 min at 20*g* before placing back into a 37 °C CO_2_ incubator. After four additional hours of coculture, cells were placed on ice and stained for flow cytometric analysis. Cells were stained with viability dye (AF700, eBioscience), Fc receptors were blocked and FACS antibodies (CD4 (clone SK3 Leu3a, APC, BD), CD8a (clone RPA-T8, PerCP-Cy5.5, BD) and mTCRβ (clone H57-597, PE, BioLegend)) were used for extracellular staining. Cells were fixed as described before and intracellularly stained with TNF (clone Mab11, BV711, BioLegend). Flow cytometric data were acquired on a BD FACS Lyric device.

### 850k methylation arrays

The 850k methylation arrays and data analysis were performed as described previously, applying Heidelberg CNS classifier version v11b4. Briefly, the Illumina Infinium HumanMethylationEPIC (EPIC) bead chip kit was used to obtain the DNA methylation status at >850,000 CpG sites (Illumina) from paraffin-embedded tissue according to the manufacturer’s instructions at the Genomics and Proteomics Core Facility of the DKFZ. MGMT promoter methylation was assessed with the use of Illumina EPIC methylation arrays based on the MGMT-STP27 model. Classification of tumors was performed with the Heidelberg classifier (www.molecularneuropathology.org). Samples were analyzed using the R (www.r-project.org) methylation pipeline ‘ChAMP’ (version 2.34.0, RRID:SCR_012891). Briefly, filtering was performed for multihit sites, single-nucleotide polymorphisms and XY chromosome-related CpGs; then, data were normalized with a BMIQ-based method. Custom scripts based on the R packages ‘minfi’ (version 1.26.2) and ‘conumee’ (version 1.14.0) were implemented for copy-number variation (CNV) profiling and visualization.^38^

### Panel sequencing

DNA from FFPE tissue was extracted on the Promega Maxwell device (Promega) following the manufacturer’s instructions. Extracted DNA was then sheared on a Covaris M220 (Covaris). DNA integrity and fragment size were determined on a Bioanalyzer 2100 (Agilent). Sequencing was performed on a NextSeq 500 instrument (Illumina) with an average coverage of 550-fold. An adapted version of the original panel consisting of a set of 170+ genes recurrently altered in brain tumors was used. For data processing, raw data were demultiplexed and converted into FASTQ format with subsequent alignment to the reference genome. For single-nucleotide variant (SNV) calling, we used SAMtools mpileup (version 1.17, RRID:SCR_002105); for indel calling, Platypus33 was used. Common sequencing artifacts were removed. Filtering was conducted for snp138 variants and exonic SNVs were included.^39^

### Statistics and reproducibility

For statistical analyses of primary endpoints, two analysis populations were defined. The safety population included all enrolled participants who had received at least one dose of IDH1-vac (SDS). The immunogenicity population (immunogenicity dataset) included all participants who could be evaluated for immunogenicity assessment. A participant was defined as evaluable if they had completed the study up to and including V07, had received at least four vaccinations through V07 and had all intended blood samples collected for immune monitoring through V07 or had received at least six of eight vaccinations and the baseline plus at least two further blood samples had been collected for immune monitoring through V12. For analysis of selected secondary variables, a molecular dataset was defined. The molecular dataset included all participants whose astrocytomas could retrospectively be defined molecularly according to CNV load, methylation class and *CDKN2A*/*B* status. Survival probabilities for OS and PFS were estimated using the Kaplan–Meier method, with the 95% CI calculated using Greenwood’s formula. For comparing participants with and without IDH1-vac-induced response in the first year, a landmark analysis was used. The Simon and Makuch method was used to illustrate the association of survival with immune response for time-dependent variables.

The median follow-up for the SDS was calculated using the reverse Kaplan–Meier method.

### Reporting summary

Further information on research design is available in the Nature Portfolio Reporting Summary linked to this article.

## Supplementary information

Reporting Summary

Supplementary Data Statistical analysis plan.

Supplementary Data Trial protocol of the NOA16 trial.

## Source data

Source Data Extended Data Fig. 1 Statistical source data.

## Data Availability

Single-cell RNA-seq data that are associated with Fig. 3 were deposited to the National Center for Biotechnology Information Sequence Read Archive under accession codes SRR12880623 and SRR12880624 and are publicly accessible. Paired αβ TCR sequence information has not been deposited for patent considerations of mutant IDH-reactive TCR motifs. TCRB sequencing data associated with Fig. 3 are available online (https://clients.adaptivebiotech.com/immuneaccess). Data that support the study findings are available to researchers upon reasonable request to the corresponding authors, if in alignment with study consent and in nonidentifiable format to protect participant privacy. Source data are provided with this paper.

## Code availability

No code was developed for this study.
