# Author Correction: *IDH1*-mutant vaccine in newly diagnosed astrocytoma: final analysis of the multicenter, single-arm, open-label, first-in-human phase 1 NOA16 trial

**DOI:** 10.1038/s43018-026-01227-x

**Published:** 2026-08-03

**Authors:** Lukas Bunse, Katharina Lindner, Antje Wick, Angelika Freitag, Lisa-Marie Lanz, Dominic Edelmann, Abigail Suwala, Michael O. Breckwoldt, Inga Harting, Felix Sahm, Richard F. Schlenk, Anita Schmitt, Oliver Schnell, Jörg Hense, Martin Misch, Dietmar Krex, Monika Denk, Juliane Walz, Joachim P. Steinbach, Andreas von Deimling, Michael Schmitt, Theresa Bunse, Ghazaleh Tabatabai, Martin Bendszus, Isabel Poschke, Wolfgang Wick, Michael Platten

**Affiliations:** 1 DKTK (German Cancer Consortium) Clinical Cooperation Unit (CCU) Neuroimmunology and Brain Tumor Immunology, German Cancer Research Center (DKFZ), Heidelberg, Germany; 2 Department of Neurology, Medical Faculty Mannheim, MCTN, University of Heidelberg, Mannheim, Germany; 3 German Cancer Consortium (DKTK), DKFZ, Core Center, Heidelberg, Germany; 4 Hertie Network of Excellence in Clinical Neuroscience, Tübingen, Germany; 5 DKFZ-Hector Cancer Institute at University Medical Center Mannheim, Mannheim, Germany; 6 Cluster of Excellence SynthImmune (EXC 3018/1) “Engineering Immune function with Synthetic Biology”, University of Heidelberg, Mannheim, Germany; 7 Immune Monitoring Unit, National Center for Tumor Diseases (NCT), Heidelberg, Germany; 8 Neurology Clinic and European Center for Neurooncology (EZN), Heidelberg University Hospital, University of Heidelberg, Heidelberg, Germany; 9 NCT Trial Management and Services, NCT, and German Cancer Research Center (DKFZ), Heidelberg, Germany; 10 Department of Neuropathology, Heidelberg University Hospital, University of Heidelberg, Heidelberg, Germany; 11 Department of Neuroradiology, Heidelberg University Hospital, University of Heidelberg, Heidelberg, Germany; 12 DKTK CCU Neuropathology, DKFZ, Heidelberg, Germany; 13 Department of Medical Oncology, Heidelberg University Hospital, University of Heidelberg, Heidelberg, Germany; 14 Department of Internal Medicine V, Heidelberg University Hospital, University of Heidelberg, Heidelberg, Germany; 15 Neurosurgery Clinic, University Hospital Erlangen, Erlangen, Germany; 16 Department of Medical Oncology, West German Cancer Center, University Hospital Essen, University of Duisburg-Essen, Essen, Germany; 17 Department of Neurosurgery, Charité Medical Center, University of Berlin, Berlin, Germany; 18 Technische Universität Dresden, Faculty of Medicine and University Hospital Carl Gustav Carus, Department of Neurosurgery, Dresden, Germany; 19 Cluster of Excellence iFIT (EXC2180) ‘Image-Guided and Functionally Instructed Tumor Therapies’, University of Tübingen, Tübingen, Germany; 20 Department of Peptide-based Immunotherapy, Institute of Immunology, University and University Hospital Tübingen, Tübingen, Germany; 21 German Cancer Consortium (DKTK) and German Cancer Research Center (DKFZ), partner site Tübingen, Tübingen, Germany; 22 Dr. Senckenberg Institute of Neurooncology, Frankfurt, Germany; 23 Department of Neurology and Neuro-Oncology, University Hospital Tübingen, Hertie Institute for Clinical Brain Research, Eberhard Karls University Tübingen, Tübingen, Germany; 24 Center for Neuro-Oncology, CCC Tübingen-Stuttgart in CCC-SW, Tübingen, Germany; 25 DKTK CCU Neurooncology, DKFZ, Heidelberg, Germany; 26 Helmholtz Institute for Translational Oncology (HI-TRON) Mainz, German Cancer Research Center, Mainz, Germany

**Keywords:** CNS cancer, CNS cancer, Tumour immunology, Peptide vaccines, Cancer

Correction to: *Nature Cancer* 10.1038/s43018-026-01199-y, published online 1 July 2026.

In the version of the article initially published, in the Abstract, the sentence “The 8-year progression-free and overall survival (OS) rates were 0.42 months (confidence interval (CI): 0.24–0.59) and 0.66 months (CI: 0.46–0.79), respectively” should have read “The 8-year progression-free and overall survival (OS) rates were 0.42 (confidence interval (CI): 0.24–0.59) and 0.66 (CI: 0.46–0.79), respectively”. This correction has been made to the HTML and PDF versions of the article.

