# Supplementary material for: IDH1-mutant vaccine in newly diagnosed astrocytoma: final analysis of the multicenter, single-arm, open-label, first-in-human phase 1 NOA16 trial
Source: Nat Cancer. 2026 Jul 1;7(8):1300–11. doi: 10.1038/s43018-026-01199-y (PMC13498463; doi:10.1038/s43018-026-01199-y)
Supplement: Supplementary file 1 [file 43018_2026_1199_MOESM1_ESM.pdf]

Corresponding author(s): Michael Platten, Lukas Bunse

Last updated by author(s): Feb 9, 2026

Reporting Summary

Nature Portfolio wishes to improve the reproducibility of the work that we publish. This form provides structure for consistency and transparency in reporting. For further information on Nature Portfolio policies, see our Editorial Policies and the Editorial Policy Checklist.

Statistics

For all statistical analyses, confirm that the following items are present in the figure legend, table legend, main text, or Methods section.

n/a	Confirmed	
☐	☒	The exact sample size (*n*) for each experimental group/condition, given as a discrete number and unit of measurement
☐	☒	A statement on whether measurements were taken from distinct samples or whether the same sample was measured repeatedly
☒	☐	The statistical test(s) used AND whether they are one- or two-sided<br>*Only common tests should be described solely by name; describe more complex techniques in the Methods section.*
☐	☒	A description of all covariates tested
☐	☒	A description of any assumptions or corrections, such as tests of normality and adjustment for multiple comparisons
☐	☒	A full description of the statistical parameters including central tendency (e.g. means) or other basic estimates (e.g. regression coefficient) AND variation (e.g. standard deviation) or associated estimates of uncertainty (e.g. confidence intervals)
☒	☐	For null hypothesis testing, the test statistic (e.g. *F*, *t*, *r*) with confidence intervals, effect sizes, degrees of freedom and *P* value noted<br>*Give P values as exact values whenever suitable.*
☒	☐	For Bayesian analysis, information on the choice of priors and Markov chain Monte Carlo settings
☐	☒	For hierarchical and complex designs, identification of the appropriate level for tests and full reporting of outcomes
☒	☐	Estimates of effect sizes (e.g. Cohen's *d*, Pearson's *r*), indicating how they were calculated

Our web collection on statistics for biologists contains articles on many of the points above.

Software and code

Policy information about availability of computer code

Data collection	single cell seq: Chromium Single Cell V(D)J Reagent kit v1 chemistry (10x Genomics), sequenced on HiSeq2500 rapid and HiSeq4000 platforms (Illumina).<br>TCRB seq: hsTCRB Kit (Adaptive Biotechnologies), sequenced on Illumina MiSeq<br>Flow Cytometry: BD FASCDiva version 8.0 (FACSAria IIu for sorting) or version 9.0 (FACSCanto II for CD8+ LIL analysis) and BD FACSuite version 1.3 (Lyric for recall assay)
Data analysis	single cell seq: processed with cellranger pipeline (version 3.1.0) with GRCh38 genome assembly (version 3.0.0, 10x Genomics).<br>TCRB seq: Data processing (demultiplexing, trimming, gene mapping) was done using Adaptive Biotechnologies´ proprietary platform.<br>Flow cytometry: FlowJo V.10.5.0.<br>exploratory analysis: GraphPadPrism V.10.6.0.

For manuscripts utilizing custom algorithms or software that are central to the research but not yet described in published literature, software must be made available to editors and reviewers. We strongly encourage code deposition in a community repository (e.g. GitHub). See the Nature Portfolio guidelines for submitting code & software for further information.

Data

Policy information about availability of data

All manuscripts must include a data availability statement. This statement should provide the following information, where applicable:

- Accession codes, unique identifiers, or web links for publicly available datasets
- A description of any restrictions on data availability
- For clinical datasets or third party data, please ensure that the statement adheres to our policy

Single cell RNA data that are associated with Figure 3 have been deposited in the NCBI Sequence Read Archive with the accession codes SRR12880623 and SRR12880624, and are publicly accessible. Paired alpha-beta TCR sequence information is not deposited for patent considerations of mutant IDH-reactive TCRs motifs. TCRB sequencing data that are associated with Figure 3 is available at https://clients.adaptivebiotech.com/immuneaccess

Research involving human participants, their data, or biological material

Policy information about studies with human participants or human data. See also policy information about sex, gender (identity/presentation), and sexual orientation and race, ethnicity and racism.

Reporting on sex and gender	62.5 % male, 37.5% female. Mean age was 40.4 (SD 8.95) years.
Reporting on race, ethnicity, or other socially relevant groupings	Inclusion into the trial was not restricted in regards to ethnicity or socially relevant groupings. The NOA16 trial, however, was a national multicenter German trial.
Population characteristics	age, gender and peripheral immune cell counts have been descriptively assessed. 71.9 % received radiotherapy (RT) plus chemotherapy with temozolomide (TMZ), 9.4 % received TMZ alone, 18.8% received RT alone. 65.6% patients had WHO grade III, 34.4% patients had WHO grade IV astrocytomas. 71.9 % of tumors located at frontal lobe. 53.1 % received complete resection, 37.5 % received subtotal resection, 9.4 % received biopsy.
Recruitment	patients were recruited at 8 trial centers in Germany, based on molecular and clinical inclusion criteria:<br>presence of a histologically confirmed IDH1R132H+ glioma (with or without measurable residual tumor after resection or biopsy) with absence of chromosomal 1p/19q co-deletion and loss of nuclear ATRX expression in the tumor tissue (subgroup of molecular astrocytoma without positive prognostic factors). In addition, inclusion criteria were:<br>patients receiced standard of care treatment (RT+TMZ, TMZ alone, or RT alone) prior to enrollment; at least 18 years old; women of child-bearing potential (WOCBP) must provide a negative pregnancy test within 72 h prior to start of IDH1 vaccination; WOCBP and their partners must use birth control method (failure rate below 1% per year).<br>exclusion criteria included: concomitant treatment with dexamethasone (or equivalent) > 2 mg/day, Karnofsky Performance Status (KPS) < 70, progressive (incl. pseudoprogression) or recurrent disease after standard of care treatment, experimental treatment of the tumor; grade 2 or higher CTCAE v4.0 laboratory values for hematology, kiver, or renal function.<br>For complete list of exclusion criteria, please refer to the study protocol.<br>Patients agreeing to trial methods are normally well-informed and motivated to comply with study procedures, which may influence results in a way of better interpretability.
Ethics oversight	The study was approved by the national regulatory authority (Paul-Ehrlich Institut) and the institutional review board at each study site (Ethikkommission), namely: Ethikkommission der Medizinischen Fakultät Heidelberg (Heidelberg), Ethikkommission Albert-Ludwigs-Universität Freiburg (Freiburg), Ethik-Kommission des Landes Berlin (Berlin), Ethik-Kommission der Medizinischen Fakultät der Universität Duisburg-Essen (Essen), Ethik-Kommission der Medizinischen Fakultät "Carl Gustav Carus" (Dresden), Ethikkommission des Fachbereichs Medizin der Goethe-Universität Frankfurt am Main (Frankfurt), Ethik-Kommission an der Medizinischen Fakultät der Eberhard-Karls-Universität und am Universitätsklinikum Tübingen (Tübingen). The study was conducted in accordance with the Good Clinical Practice guidelines of the International Conference on Harmonisation. All participants provided written signed informed consent.

Note that full information on the approval of the study protocol must also be provided in the manuscript.

Field-specific reporting

Please select the one below that is the best fit for your research. If you are not sure, read the appropriate sections before making your selection.

☒ Life sciences ☐ Behavioural & social sciences ☐ Ecological, evolutionary & environmental sciences

For a reference copy of the document with all sections, see nature.com/documents/nr-reporting-summary-flat.pdf

Life sciences study design

All studies must disclose on these points even when the disclosure is negative.

Sample size	Sample size estimation was primarily based on the accuracy requirements for the primary endpoint immune response (responder rate) to the IDH1 peptide vaccine. Sample size was adjusted for non-evaluable patients. Estimation that 70% of patients evaluable for immunogenicity testing will be evaluable for all time points (Macdonald D, Cascino T, Schold SJ, et al: Response criteria for phase II studies of supratentorial

	malignant glioma. J Clin Oncol 8:1277-1280, 1990.) 21 patients sufficient for immunogenicity testing with all time points, i.e. 30 evaluable patients to be enrolled. due to expected dropout rate of 20% (due to progression or other reasons), 39 patients to be recruited.
Data exclusions	Of all 32 patients treated (= safety data set), 2 patients were excluded from immunogenicity testing, because they were not evaluable, because not enough time points were eligible to immunogenicity testing (Immungenicity Data Set). A patient was pre-defined to be evaluable if study completed until and incl. visit 7, received at least 4 vaccinations and all blood samples collected for immunogenicity testing, OR received 6 vaccinations and baseline plus 2 blood samples collected for immunogenicity testing. Molecular testing was done retrospectively. For n=24 patients, sufficient material was available for additional molecular testing
Replication	Findings concerning patient outcomes and immunogenicity at defined time points, and all related analyses using patient blood samples or derivatives thereof cannot be reproduced due to sample limitations. TCR testing was reproduced at least three times with similar outcome.
Randomization	Phase 1 study, i.e. All patients received IDH1 vaccination (IDH1-vac). In addition, they received standard of care treatment prior to enrollment as decided by the local investigator and the patient. 3 types of standard of care treatment resulted in 3 treatment groups, all receiving the exact same trial related intervention.<br>For TCRB deep seq and single cell RNA/TCR-seq, PBMC and tissue samples were selected based on availability, respectively.<br>In flow cytometry, cells were allocated to different stainings (full stain panels, staining controls) randomly.
Blinding	Single arm, open label trial, i.e. neither patients nor clinical nor immunogenicity investigators were blinded concerning IDH1 vaccination (=trial related intervention). With respect to standard of care treatment groups, immunogenicity investigators were blinded. All primary endpoint analyses were done in a blinded fashion. Exploratory analyses e.g. immunological phenotyping were performed non-blinded with respect to immune response detectable in the sample, because samples for these analyses were selected based on immune response.

Reporting for specific materials, systems and methods

We require information from authors about some types of materials, experimental systems and methods used in many studies. Here, indicate whether each material, system or method listed is relevant to your study. If you are not sure if a list item applies to your research, read the appropriate section before selecting a response.

Materials & experimental systems

n/a	Involved in the study
☐	☒ Antibodies
☒	☐ Eukaryotic cell lines
☒	☐ Palaeontology and archaeology
☒	☐ Animals and other organisms
☐	☒ Clinical data
☒	☐ Dual use research of concern
☒	☐ Plants

Methods

n/a	Involved in the study
☒	☐ ChIP-seq
☐	☒ Flow cytometry
☐	☒ MRI-based neuroimaging

Antibodies

Antibodies used	Flow cytometry antibodies for immunophenotyping:<br>anti-CD3-FITC (clone UCHT1, cat # 300452), anti-CD4-Alexa Fluor700 (clone RPA-T4, cat # 300526), anti-CD8-PerCP (clone RPA-T8, cat # 301030), anti-CD11b-BV510 (clone M1/70, cat # 101263), anti-HLA-DR-PE-Cy7 (clone L243, cat # 307616), anti-CD14-BV711 (clone M5E2, cat # 301838), anti-CD16-PE/Dazzle594 (clone 3G8, cat # 302054), anti-CD25-BV605 (clone BC96, cat # 302632), anti-CD33-APC (clone P67.6, cat # 366606), anti-CD127-BV421 (clone A019D5, cat # 351310), anti-Foxp3-PE (clone 206D, cat # 320108) (all BioLegend), anti-CD45-eFluor450 (clone 2D1, cat # 48-9459-42, eBioscience), anti-CD3-PE (clone HIT3a, cat # 300308, BioLegend), anti-CD3-BV510 (clone HIT3a, cat # 564713), anti-CD4-BV605 (clone SK3, cat # 566908), anti-CD8-APC-H7 (clone SK1, cat # 560179), anti-IFNgamma-BV421 (clone 4S.B3, cat # 564791), all BD Biosciences, anti-TNFalpha-APC (clone MAb11, cat # 502912, Biolegend), anti-IL17-PE (clone N49-653, cat # 560486), anti-IL4-PerCP-Cy5.5 (clone 8D4-8, cat # 561234), anti-CD25-BV711 (clone 2A3, cat # 563159), anti-CD127-FITC (clone HIL-7R-M21, cat # 560549), anti-FoxP3-PE (clone 259D/C7, cat # 560046), anti-IL10-APC (clone JES3-19F1, cat # 554707) , anti-CD3-BV510 (clone HIT3a, cat # 564713) (all BD Biosciences), anti-CD8-PerCP-Cy5.5 (clone RPA-T8, cat # 45-0088-42), anti-TNFalpha-APC (clone MAb11, cat # 17-7349-82), and anti-IFNgamma-eFluor450 (clone 4S.B3, cat # 48-7319-42) (all ebioscience).<br><br>ELISA antibodies:<br>mouse anti-IDH1R132H (clone H09, cat # DIA-H09, Dianova), sheep anti-mouse IgG-HRP (polyclonal secondary, cat # NXA931, Amersham) and goat anti-human IgG-Fc-HRP (polyclonal secondary, cat # A80-104P, Bethyl Laboratories, Inc.).
Validation	all antibodies were titrated prior to use as advised by supplier in the material data sheet or used according to manufacturer's instructions, and, for flow cytometry, scaled up according to cell numbers.<br>Flow cytometry antibodies: each lot is quality control tested by extra- or intra-cellular flow cytometry using positive control samples and appropriate isotype control stainings.<br>anti-IDH1R132H (clone H09, cat # DIA-H09, Dianova): approved for in vitro diagnostic use with IHC in Europe.<br>secondary ELISA antibodies: Bethyl: By immunoelectrophoresis and ELISA this antibody reacts specifically with human IgG. Cross reactivity with IgM, IgA and light chains is less than 0.1%. This antibody may cross react with IgG from other species. Amersham: Horseradish peroxidase (HRP) conjugated antibodies are highly species specific antibodies optimized for use with Amersham ECL Western blotting detection reagents.

Clinical data

Policy information about clinical studies
All manuscripts should comply with the ICMJE guidelines for publication of clinical research and a completed CONSORT checklist must be included with all submissions.

Clinical trial registration	NCT-2013-0216
Study protocol	not publicly accessible. all relevant information can be found on ClinicalTrials.gov
Data collection	Patients were recruited at 8 sites across Germany: Heidelberg (14 patients), Freiburg (2 patients), Berlin (4 patients), Essen (3 patients), Dresden (3 patients), Frankfurt (3 patients), Tuebingen (4 patients). Screening was between July 2015 and September 2016. Enrollment was between September 2015 and October 2016. Last patient completed the study in September 2017. Endpoint-related Immunogenicity analyses and related analyses were completed in June 2020. Follow-up data cut-off for final analysis was Feb 2025. After 2020, this was done by 6-month questionnaire-based data retrieval from trial centers.
Outcomes	primary objectives were safety and tolerability; and immunogenicity of the IDH1 vaccine.<br>Saftey measures were assessed by medical review at each visit (every 2 or 4 weeks for 8 visits during vaccinations; 4, 8, and 12 weeks after last vaccination), incl. reporting of adverse events (AEs), concomitant medications. All AEs graded according to National Cancer Institute Common Terminology Criteria for Adverse Events (CTCAE) version 4.0. Primary endpoint regime limiting toxicity (RLT) defined as: - any injection site reaction of CTCAE grade 4; any injection site reaction of CTCAE grade 3 that persists after two weeks; any other hypersensitivity, anaphylaxis or local allergic reaction ≥ CTCAE grade 3; brain edema (CTCAE grade 4); autoimmunity ≥ CTCAE grade 3; ≥ CTCAE grade 3 toxicity to organs other than the bone marrow, but excluding grade 3 nausea, grade 3 or 4 vomiting in patients who have not received optimal treatment with antiemetics, grade 3 or 4 diarrhea in patients who have not received optimal treatment with antidiarrheas, grade 3 fatigue; death; that is definitely/certainly, probably, or possibly related to the IDH1 vaccine. patients with RLT removed from treatment, no dose-de-escalation but skipping of vaccines allowed due to AEs.<br>Immunogenicity endpoint (induction of presence of IDH1-reactive T cells or binding antibodies) was assessed by ELIspot and ELISA from blood PBMC and serum collected at baseline, 2 or 4 weeks after each vaccination, and 12 and 24 weeks after last vaccination. T cell immunogneicity was defined as specific (negative control subtracted) spot forming unit SFU) count of at least 50 at any time point but baseline if no spontaneous response detectable. In case of spontaneous response, SFU post-IDH1-vaccination were defined to be at least 3-fold above baseline. For antibodies, optical density of at least 5-fold above negative control at any time point was defined positive.<br>secondary objectives were (1) to seek evidence of immunogenicity by assessing the IDH1R132H-specific T-cell and antibody response measured by IFN-γ ELISpot and ELISA, respectively, at all time points of blood withdrawal; (2) to evaluate clinical outcome by assessing the progression-free survival (PFS) and overall response rate (ORR) according to the response evaluation criteria defined as follows: ORR, defined as the proportion of patients showing complete response (CR), partial response (PR) or stable disease (SD) at end of study (EOS) compared to the baseline value for ORR under trial drug. ORR analysis will be based on the central disease assessment according to the RANO criteria; and (3) to analyze the association between immunogenicity and the clinical outcome parameters.

Plants

Seed stocks	n/a
Novel plant genotypes	n/a
Authentication	n/a

Flow Cytometry

Plots

Confirm that:

- ☒ The axis labels state the marker and fluorochrome used (e.g. CD4-FITC).
- ☒ The axis scales are clearly visible. Include numbers along axes only for bottom left plot of group (a 'group' is an analysis of identical markers).
- ☒ All plots are contour plots with outliers or pseudocolor plots.
- ☒ A numerical value for number of cells or percentage (with statistics) is provided.

Methodology

Sample preparation	peripheral immune monitoring of PBMC: PBMC were isolated by ficoll gradient centrifugation from heparin blood, frozen in 50% freezing medium A (60% X-Vivo 20 + 40% FCS) and 50% medium B (80% FCS + 20% DMSO) and stored in liquid nitrogen

	at -140°C until analysis.<br>recall assay of PBMC: PBMC were thawn, rested for 4 h in X-Vivo medium, and seeded into 96-well U-bottom plates. 2x106 PBMCs were stimulated with 2 µg peptide per well using IDH1R132H(p123-142), MOG(p35-55) as negative control, or CEFT peptide pool (concentration, jpt) as positive control for 2 h before adding 10 µg/ml brefeldin A (Sigma-Aldrich, order no. B6542) and 1x GolgiStop (BD Bioscience). Cells were incubated additional 12 h.
Instrument	peripheral immune monitoring of PBMC: Attune Nxt (Thermo Fisher Scientific); recall assay of PBMC: Lyric (BD)
Software	collection: Attune Nxt software version 2.7 (Thermo Fisher Scientific) (peripheral immune monitoring); BD FACSDiva software version 9.0; BD FACSuite version 1.3 (recall assay).<br>analysis: FlowJo version 10.5.0.
Cell population abundance	relevant post-sorted cell fractions, i.e. CD3+ T cells, were subjected to scRNA sequencing. Based on RNA expression signatures, after further removal of stressed cells (by expression of heat shock proteins), 99.42% of cells were T cells. Non-T cells were excluded from analysis. The remaining cells were used for TCR repertoire analyses.
Gating strategy	peripheral immune monitoring of PBMC:<br>gated on lymphocytes based on size and granularity (FSS vs SSC) --> gated on single cells --> gated on live cells, i.e. dead stain negative cells --> gated on: A. CD3+ --> CD8+/CD4+ --> CD4+ gated on CD25+ CD127 --> gated on FoxP3+ cells = Treg, and B. CD33+ --> gated on a. CD11b+ HLA-DR+ --> CD14-/CD16- cells = monocytes, and b. CD11b+/HLA-DR- --> CD14+ cells = Mo-MDSC<br>recall assay:<br>gated on lymphocytes based on size and granularity (FSS vs SSC) --> gated on single cells --> gated on live cells, i.e. dead stain negative cells --> gated on CD3+ --> gated on A. CD4- CD8+ --> gated on IFNgamma+ and TNFalpha+, and B. CD4+ CD8- --> gated on IFNgamma+ and TNFalpha+ (Figure 4)

☐ Tick this box to confirm that a figure exemplifying the gating strategy is provided in the Supplementary Information.

Magnetic resonance imaging

Experimental design

Design type	Structural MRI
Design specifications	At least 6 MRI scans were acquired for each patient (pre study; clinical screening, visit 7,10,12,13), follow up MRIs were conducted at trial centers and external centers. Sequences were performed as required for RANO / RANO2.0 in the follow-up. The specifications listed below are specific to on trial scans.
Behavioral performance measures	MRI used solely for diagnosis of disease progression according to RANO.

Acquisition

Imaging type(s)	T2-w; FLAIR; contrast enhanced T1-w
Field strength	3 Tesla
Sequence & imaging parameters	Parameter T2-w (TSE); TE: 88; TR: 5.280; Flip angle: 180; FOV: 230 × 230; Matrix size: 256 × 173; Slice thickness: 5; No. of averages 1; In-plane resolution 0.7 Orientation Axial (2D) Duration 1:15 (min:s)<br><br>Parameter FLAIR; TE: 135; TR: 8.500; Flip angle: 170; FOV: 230 x 170; Matrix size: 320 × 216; Slice thickness: 5 No. of averages 1 In-plane resolution: 1 Orientation Axial (2D) Duration 2:52 (min:s)<br><br>T1 (mpRAGE): TE: 3.57; TR: 1.770; Flip angle: 15; FOV: 256 × 256; Matrix size: 320 × 272; Slice thickness: 1 No. of averages: 1; In-plane resolution 1; Orientation sagittal(3D); Duration 3:27 (min:s)
Area of acquisition	whole brain scan

Diffusion MRI ☐ Used ☒ Not used

Preprocessing

Preprocessing software	n/a
Normalization	n/a
Normalization template	n/a
Noise and artifact removal	n/a
Volume censoring	n/a

Statistical modeling & inference

Model type and settings	n/a
Effect(s) tested	n/a
Specify type of analysis:	☐ Whole brain ☐ ROI-based ☐ Both
Statistic type for inference (See Eklund et al. 2016)	n/a
Correction	n/a

Models & analysis

n/a	Involved in the study
☒	☐ Functional and/or effective connectivity
☒	☐ Graph analysis
☒	☐ Multivariate modeling or predictive analysis
