# Supplementary material for: IDH1-mutant vaccine in newly diagnosed astrocytoma: final analysis of the multicenter, single-arm, open-label, first-in-human phase 1 NOA16 trial
Source: Nat Cancer. 2026 Jul 1;7(8):1300–11. doi: 10.1038/s43018-026-01199-y (PMC13498463; doi:10.1038/s43018-026-01199-y)
Supplement: Supplementary file 2 [file 43018_2026_1199_MOESM2_ESM.pdf]

STATISTICAL ANALYSIS PLAN

Final 1.0

Targeting IDH1R132H in WHO grade III IV IDH1R132H-mutated gliomas by a peptide vaccine

# – a Phase I safety, tolerability and immunogenicity multicenter trial

(NOA-16)

**Clinical Trial Code:**	NCT-2013-0216
**EudraCT No.:**	2014-000503-27
**Clinical Phase:**	Phase I, first-in-man
**Version and date:**	Final version 3.0, 12.04.2016

Sponsor:

Ruprecht-Karls-University Heidelberg, Medical Faculty
- represented in law by Universitätsklinikum Heidelberg and its Commercial Director Ms. Irmtraut Gürkan -
Im Neuenheimer Feld 672
69120 Heidelberg, Germany
Phone: +49 (0)6221 / 56-7002
Fax: +49 (0)6221 / 56-4888
E-Mail: Irmtraut.Guerkan@med.uni-heidelberg.de

Coordinating Investigator:

Prof. Dr. Michael Platten
Neurology Clinic
University Hospital Heidelberg
Neurooncology Program at the NCT
Im Neuenheimer Feld 400
69120 Heidelberg, Germany
Phone: +49 (0)6221 / 56-6804
Fax: +49 (0)6221 / 56-7554
E-Mail: michael.platten@med.uni-heidelberg.de

Approvals

**Author and Project Biometrician:**		
Irini Karapanagiotou-Schenkel	*Date (dd/mm/yyyy)*	*Signature*
**Coordinating Investigator:**		
Prof. Dr. med. M. Platten	*Date (dd/mm/yyyy)*	*Signature*

Signature above indicates approval of this plan, for the analysis and reporting of this study.

Table of Content

LIST OF ABBREVIATIONS 5
1. INTRODUCTION 8
 1.1. Amendments from Previous Version(s) 8
 1.2. Study Objectives 8
 1.3. Study Design 9
 1.4. Study Drug, Study Dosing and Treatment Groups 9
 1.5. Study Population 9
 1.6. Study Period and Visit Window Definitions 9
 1.7. Hypotheses and Decision Rules 10
 1.8. Sample Size Justification 10
 1.9. Randomization and Blinding 10
2. POPULATIONS OF ANALYSIS 10
 2.1. Screened Population 10
 2.1. Safety Population 11
 2.2. Immunogenicity Population 11
 2.3. Sub-group Definitions 11
 2.4. Treatment Misallocations (n.a.) 11
 2.5. Protocol Deviations 11
 2.6. Definition and use of Visits Windows in reporting 12
3. STUDY ENDPOINTS AND COVARIATES 12
 3.1. Primary Endpoints 12
  3.1.1. Safety endpoint 12
  3.1.2. Immunogenicity Endpoint 13
 3.2. Secondary Endpoints 13
 3.3. Safety and Tolerability Endpoints 14
 3.4. Translational Research Endpoints .... **Fehler! Textmarke nicht definiert.**
 3.5. Covariates 14
4. STATISTICAL METHODOLOGY 15
 4.1. General Methodology 15
 4.2. Statistical Analysis 15
  4.2.1. Analysis of the Primary Endpoints 15
  4.2.2. Analysis of the Secondary and exploratory endpoints 15
  4.2.3. Analysis of the exploratory endpoints .... **Fehler! Textmarke nicht definiert.**

4.2.4. Safety Data Analysis 16
4.2.5. Further Analyses 17
4.3. Statistical/Analytical Issues 17
4.3.1. Adjustments for Covariates....17
4.3.2. Multiple Comparisons/Multiplicity....17
4.3.3. Handling of Dropouts, Missing Data, and Outliers....17
4.3.4. Center Pooling Method and Handling Center Effects....17
4.3.5. Interim Analyses and Data Monitoring 18
5. STATISTICAL REPORTING....18
5.1. Study Patients and Data Sets Analyzed 18
5.2. Demographic and Patient Characteristics....18
5.3. Evaluation of Treatment Compliance and Exposure 18
5.4. Analysis of the primary immunogenitity Endpoint....19
5.4.1. Autoimmunity....19
5.4.2. (Reccurrent) Tumor Tissue....19
5.4.3. Tumor Measurement ....**Fehler! Textmarke nicht definiert.**
5.5. IDH1 Peptide Vaccination/ Imiquimod/Aldara 19
5.6. Analysis of the primary Safety Endpoint 20
5.6.1. Regime-Limiting Toxicity (RLT)....20
5.7. Analysis of Further Safety Endpoints....20
5.7.1. Adverse Events 20
5.7.2. Clinical Laboratory Evaluation 20
5.7.3. Vital Signs, Physical Findings and Other Observations Related to Safety....20
5.8. Analysis of the Clinical Outcomes 20
5.9. Translational Research....**Fehler! Textmarke nicht definiert.**
5.10. Interim Analyses and Safety Monitoring Analyses**Fehler! Textmarke nicht definiert.**
6. REPORTING CONVENTIONS ANDPLANNED DATA DISPLAYS 21
7. REFERENCES....21
8. APPENDICES 21

LIST OF ABBREVIATIONS

2-HG	R-2-Hydroxy-Glutarate
2-HG-MRS	2-Hydroxy-Glutarate Magnetic Resonance Spectroscopy
90% (95%) CI	90% (95%) confidence interval
AE	Adverse Event
ADL	Activity of Daily Living
AG	Grade III (anaplastic) gliomas
ALT	Alanine Amino Transferase, also known as SGPT
ANC	Absolute Neutrophil Count
AST	Aspartate Amino Transferase, also known as SGOT
ATC	Anatomisch Therapeutisch Chemisches Klassifikationssystem
AMG	German Drug Law (Deutsches Arzneimittelgesetz)
ATRX	α-thalassemia/mental-retardation-syndrome-X-linked
BDSG	Bundesdatenschutzgesetz
BUN	Blood Urea Nitrogen
CI	Coordinating Investigator (LKP)
CRF	Case Report Form
CRP	C Reactive Protein
CT	Computer Tomography
CTCAE	Common Toxicity Criteria for Adverse Events
DBL	Data Base Lock
DKTK	German Cancer Consortium (Deutsches Konsortium für Translationale Krebsforschung)
DMC	Data Monitoring Committee
DMSO	Dimethylsufoxide
DVP	Data Validation Plan
EC	Ethics Committee
ECG	Electrocardiogram
ELISA	Enzyme Linked Immunosorbent Assay
ELISpot	Enzyme Linked Immuno Spot Assay
EMA	European Medicines Agency
EOS	End of Study
EOT	End of Treatment
ESF	Eligibility Screening Forms
FDA	US Food and Drug Administration
FFPE	Formalin-fixed Paraffin-embedded
FPFV	First Patient First Visit
fT4	Tetrajodthyronin / Thyroxin
fT3	Trijodthyronin
FU	Follow-up

GB	Glioblastoma
GCP	Good Clinical Practice
GCP-V	Good Clinical Practice Ordinance (GCP-Verordnung)
GGT	Gamma Glutamyl Transpeptidase
GM-CSF	Granulocyte macrophage colony-stimulating factor
GMP	Good Manufacturing Practice
HBV	Hepatitis B Virus
HCV	Hepatitis C Virus
HIV	Human Immunodeficiency Virus
H-score	Immonohistochemistry Score/Histo Score
IB	Investigator´s Brochure
ICH	International Conference on Harmonization
ICD	International Classification of Disease
i.d.	intradermal
IDH1	Isocitrate Dehydrogenase Type 1
IDH1R132H	IDH1 with amino acid exchange from arginine to glutamine at position 132
IFA	Incomplete Freund's Adjuvant
IIT	Investigator Initiated Trial
IMP	Investigational Medicinal Product
IMPD	Investigational Medicinal Product Dossier
INN	International Nonproprietary Name
INR	International Normalized Ratio
IR	Immunogenic response
ISF	Investigator Site File
ISRCTN	International Standard Randomised Controlled Trial Number
ITT	Intention To Treat
IUD	intrauterine device
IUS	intrauterine system
KKS	Coordination Center for Clinical Trials (Koordinierungszentrum für Klinische Studien)
KPS	Karnofsky Performance Status
LDH	Lactate Dehydrogenase
LGG	Grade II (low grade) gliomas
LKP	Coordinating Investigator according to AMG (Leiter der Klinischen Prüfung)
LPFV	Last Patient First Visit
LPLV	Last Patient Last Visit
MCH	Mean Corpuscular Hemoglobin
MCHC	Mean Corpuscular Hemoglobin Concentration
MCV	Mean Corpuscular Volume

MedDRA	Medical Dictionary for Regulatory Activities
MMSE	Mini Mental Status Examination
MRI	Magnetic Resonance Imaging
MRS	Magnetic Resonance Spectroscopy
n.a.	Not applicable
ORR	Overall Response Rate
PFS	Progression Free Survival

1. INTRODUCTION

The purpose of this SAP is to outline the planned analyses and to support the completion of the statistical report. The planned analyses identified in this SAP will be included in future publications. Any post-hoc or unplanned analyses not identified in this SAP will be clearly identified in the respective final study report and/or future manuscripts.

The structure and content of this SAP provides sufficient detail to meet the requirements identified by the International Conference on Harmonisation of Technical Requirements for Registration of Pharmaceuticals for Human Use (ICH).

The following documents were reviewed in preparation of this SAP:

- Study Protocol, Version Final 3.0, dated 12.04.2016
- Case report forms (CRFs), Final Version
- Data Management Plan (DMP)
- Final Version DMC-Reports
- ICH Guidelines E3 “Structure and Content of Clinical Study Reports"
- ICH Guidelines E9 “Statistical Principles for Clinical Trials".

The reader of this SAP is encouraged to also read the study protocol for details on the conduct of this study, the operational aspects of clinical assessments and timelines of treatment in this study.

1.1. Amendments from Previous Version(s)

This is the first SAP Version. There are no amendments from previous version.

1.2. Study Objectives

The primary objectives of this trial are:

- to determine safety and tolerability of repeated fixed dose vaccinations of the IDH1 peptide vaccine administered with topical imiquimod (Aldara®). Primary safety endpoint is the Regime-Limiting Toxicity (RLT). The definition is given in section 3.1.1.
- to assess immunogenicity of the IDH1 peptide vaccine and hence demonstrate "proof of principle" for the vaccination strategy. The primary immunogenicity endpoint is the presence of an IDH1R132H-specific T-cell and/or antibody response as dichotomous variable. The detailed definition is given in section 3.1.2.

Secondary Objectives are

- to seek evidence of immunogenicity by assessing the IDH1R132H-specific T-cell and antibody response at visits 3, 5, 7, 10, 12, and 13,
- to assess progression-free survival (PFS) and overall response rate (ORR), and
- to analyze the association between immunogenicity and the clinical outcome parameters.

Translational Research

It was planned to perform analyses for the translational research of the following assessments:

- to determine magnet resonance spectroscopy (MRS) parameters including R-2-hydroxyglutarate (2 HG) for detection of intra-tumoral IDH1R132H enzyme activity (only if the patient has measurable residual disease, if local neuroradiology has implemented the method, and if baseline MRS data are available for visit 2)
  This parameter could not be performed due to insufficient results regarding the sensitivity and specificity analyses of the available sequences.
- to characterize the IDH1R132H-reactive T cell and antibody subtypes
- to relate immunogenicity to the HLA type
- to relate immunogenicity and clinical outcome to the presence of IDH1R132H DNA in the peripheral circulation
- to analyze IDH1R132H immunoreactivity in recurrent tumors, if reoperation or biopsy is clinically indicated and tissue is available, and if local laboratory has implemented a protocol for sample processing
- to assess IDH1R132H mutation status in recurrent tumors, if reoperation or biopsy is clinically indicated and tissue is available

The Translational research analysis will be performed in the Central Immune Laboratory Unit of the German Cancer Research Center and a separate report about this analysis will be attached to the final study report.

1.3. Study Design

The study NOA-16 is a non-controlled, open-label, single arm, multicenter, national, first-in-man phase I trial to analyze safety, tolerability and immunogenicity of repeated doses of the IDH1 peptide vaccine in patients with gliomas carrying the IDH1R132H mutation.

1.4. Study Drug, Study Dosing and Treatment Groups

Within this trial, the IDH1 peptide vaccine – a 20mer peptide encompassing the IDH1R132H-mutated region emulsified in Montanide® – is administered subcutaneously in combination with topical imiquimod (5%, Aldara®). The vaccine is administered in weeks 1, 3, 5, 7, 11, 15, 19 and 23 (visits 3 to 10).

1.5. Study Population

The patient population is molecularly defined and includes IDH1R132H mutant grade III and IV gliomas without co-deletion of 1p/19q and with loss of alpha-thalassemia/mental retardation syndrome X-linked (ATRX) expression. Patients must have received radiotherapy alone (treatment group 1), 3 cycles of chemotherapy with temozolomide (TMZ, treatment group 2) or combined radiochemotherapy with TMZ (treatment group 3) prior to enrollment.

1.6. Study Period and Visit Window Definitions

The duration of the trial for each patient was expected to be maximum 62 weeks including molecular and clinical screening, vaccination period as well as end-of-treatment (EOT), Safety follow-up and end-of-study (EOS) visit.

End-of-treatment (EOT) visit, Safety follow-up and End-of-study (EOS) were to be performed 4, 12 and 24 weeks after last vaccination, respectively. The timing of scheduled assessments is shown in the trial schedule and the flow chart of the study protocol.

1.7. Hypotheses and Decision Rules

In this trial early stopping rules have been implemented to detect unacceptable rates of toxicities (frequency of RLTs) or unexpected shortening of PFS. The critical boundaries to stop the recruitment due to an inacceptable rate of toxicities were calculated by means of computer simulations during the planning phase. The stopping rule was based on the number of RLTs among all patients enrolled. These numbers are continuously monitored by the NCT Trial Center.

To ensure that the therapy does not negatively affect the course of disease, interim analyses were to be performed to detect unexpected shortening of PFS. These analyses are carried out once 10 (and if applicable, 20 and 30) patients have reached either 12 months of follow-up or an event, whichever comes first. Interim analyses were to be performed only on the condition that not all 39 study patients have reached a follow-up of 12 months prior to the analysis (for otherwise any decision to stop the accrual or treatment of study patients would be futile). Shortening of PFS was defined as an observed decrease of at least 10 % in the estimated 12-months PFS rate compared to the anticipated value of 70.7% derived from previous studies.

If an unacceptable rate of toxicities or shortening of PFS was observed, recruitment was to be interrupted and the data monitoring committee was to be informed immediately to provide advice to the Coordinating Investigator concerning the continuation or discontinuation of recruitment and/or treatment.

1.8. Sample Size Justification

Sample size estimation is primarily based on the accuracy requirements for the primary endpoint ‘immune response’ to the IDH1 peptide vaccine. In a second step it has been verified that the intended patient number also fulfills the accuracy requirements for the assessment of the RLT.

To be able to assess safety, tolerability and immunogenicity of the peptide vaccine, 30 evaluable patients (maximum 39 patients in total) were to be enrolled into the trial. The sample size was to be adjusted for non-evaluable patients (for definition refer to section 5.1 of the study protocol), except for drop-outs due to RLT. The corresponding dropout rate was expected to be 20% and thus, 39 patients have been recruited. The rate of molecular screening failures for inclusion into this study was expected to be about 55%. Based on this assumption the necessary number of molecularly screened patients was 87.

Recruitment and treatment of patients was performed in 8 trial sites, which are part of the German Cancer Consortium (DKTK) and/or the Neurooncology Working Group of the German Cancer Society (NOA).

1.9. Randomization and Blinding

n.a. This is a non-controlled open-label trial.

2. POPULATIONS OF ANALYSIS

2.1. Screened Population

This population includes treated patients as well as patients who were screened only or enrolled in the study, but have not received any amount of the study treatment. For patients who were screened, but not included into the study only their demographic data

(Screening ID, gender, age, Tumor Diagnosis) and the reason for not inclusion into the study will be tabulated, but will not be included in any statistical analysis.

2.1. Safety Population

The Safety population includes all enrolled patients who have taken at least one dose of trial treatment. This is the primary analysis set for evaluating patient characteristics, study administration, and all efficacy and safety endpoints. Unless otherwise noted, by-visit efficacy analyses will be performed for the safety analysis set.

2.2. Immunogenicity Population

This population includes all patients who are evaluable regarding the immunogenicity. A patient is defined as evaluable if:

- he/she has completed the study up to and including study visit 7, has received at least 4 vaccinations through visit 7 and has all intended blood samples collected for immune monitoring through visit 7, OR
- he/she has received at least 6 of 8 vaccinations, and baseline plus two further blood samples collected for immune monitoring through visit 12.

Non-evaluable patients were to be replaced for assessment of immunogenicity, except for patients leaving the study early due to RLT.

2.3. Sub-group Definitions

The trial population will comprise 3 sub-groups based on the standard treatment the patients have received prior to enrollment:

- Treatment Group 1: radiotherapy alone,
- Treatment Group 2: 3 cycles of chemotherapy with TMZ alone, and
- Treatment Group 3: combined radiochemotherapy with TMZ.

In treatment group 1 vaccination treatment was to be done alone starting 4-6 weeks post radiotherapy. In treatment groups 2 and 3 vaccination treatment was to be done in parallel with TMZ chemotherapy starting at day 10 of the 4th cycle of the TMZ monotherapy (treatment group 2) or at day 10 of the 1st TMZ cycle post concomitant radiotherapy (treatment group 3; for details see section 7.1 and the flowchart on p. 18 of the study protocol).

2.4. Treatment Missallocations (n.a.)

n.a. This is a non-controlled, single-arm trial.

2.5. Protocol Deviations

All relevant deviations related to study inclusion or exclusion criteria, conduct of the study, patient management, or patient assessment will be described. Major protocol violations include any concomitant medication that may confound study results or any non-compliance issues raised by the investigator prior to the blind review meeting using the sample listing of protocol deviations (ref. Appendix A1) and will be discussed prior to database closure at the blind data review meeting.

For this purpose the list of protocol deviations which was prepared during the study period as part of the DMC reporting (s. DMC Report Appendix 1, section 3) will be used, but some adjustment to final data may be necessary.

2.6. Definition and use of Visits Windows in reporting

Scheduled visits (s. also Trial schedule) and visit windows are shown in table 1 below:

Table 1: Visits and Visit Windows

Visit (v)	Visit Windows
**Molecular Screening, v1**	Days: -3 to day -1
**Clinical Screening, v2**	Days: -16 to -2
**Vaccination, v3 to v10**	Days: 1, 15±3, 29±3, 43±3, 71±3, 99±3, 127±3, 155±3
**EOT** (End of Treatment)**, v11**	Day 183±3
**Safety Follow-up, v12**	Day 239±3
**EOS (**End of Study)**, v13**	Day 323±3

Variables observed at particular visit windows will be summarized by visit, regardless of the actual time point the observation was performed. The conventions about the allowable range to be used for categorizing dated assessments under the nominal visits for the purpose of reporting is to be compiled on the blinded data review meeting. A list of all visit deviations will be prepared (ref. SAP Appendix A1) and will be discussed prior to database closure, at the blind data review meeting.

3. STUDY ENDPOINTS AND COVARIATES

3.1. Primary Endpoints

3.1.1. Safety endpoint

Primary safety endpoint is the **Regime-Limiting Toxicity (RLT)** defined as

- any injection site reaction of CTCAE grade 4
- any injection site reaction of CTCAE grade 3 that persists after two weeks
- any other hypersensitivity, anaphylaxis or local allergic reaction ≥ CTCAE grade 3
- brain edema (CTCAE grade 4)
- autoimmunity ≥ CTCAE grade 3
- ≥ CTCAE grade 3 toxicity to organs other than the bone marrow, but excluding
    - grade 3 nausea
    - grade 3 or 4 vomiting in patients who have not received optimal treatment with anti-emetics
    - grade 3 or 4 diarrhea in patients who have not received optimal treatment with anti-diarrheas
    - grade 3 fatigue

- death

that is definitely/certainly, probably, or possibly related to the administration of the IMP.

Patients who experience RLT were to be removed from trial treatment. Dose de-escalation of the trial agents was not allowed, but vaccination might to be skipped because of AEs (for details see section 6.4.2.1 of the study protocol).

Transient fever (> 38 °C) and acute infections were not defined as RLTs, but vaccinations should be skipped in such cases until the patient fully recovered (refer to section 6.4.2.1 of the study protocol).

3.1.2. Immunogenicity Endpoint

Determination of the immunogenicity endpoint was to be based on the assessment of the patient's IDH1R132H-specific T-cell and antibody response.

Immunogenic response (IR) is evaluated as a dichotomous variable defined as an IDH1R132H-specific T-cell (ELISpot) and/or antibody response (ELISA) at any time during the trial measured by IFN-γ ELISpot and peptide-coated ELISA, respectively.

Definition of the dichotomous immunogenic response (IR):

For any time during the observation period,

IR='YES", if either EliSpot ="YES" or ELISA (all peptides="YES") ="YES"

Depending on patient immunologic status at visit 3, immunogenic response is defined as follows:

- For patients **without** IDH1R132H-specific T-cell and/or antibody positivity at visit 3 prior to the first vaccination (i.e. without spontaneous immune response):
  IR = IDH1R132H-specific T-cell and/or antibody positivity at any of the visits 5, 7, 10, 12, and 13.
- For patients **with** spontaneous immune response, i.e. IDH1R132H-specific T-cell and/or antibody positivity at visit 3 prior to the first vaccination
  IR = an at least 3-fold increase of the IDH1R132H-specific T-cell and/or antibody value at any of the visits 5, 7, 10, 12, and 13 compared to the baseline value (visit 3).

Cutoffs for IDH1R132H-specific T-cell and antibody positivity were evaluated using sera of patients with IDH1R132H-mutated or wild-type glioma and healthy donors:

- For IFN-γ ELISpot a cut-off of 50 IFN-γ spots after substraction of baseline (MOG) was defined by stimulating PBMC with IDH1 p123-142 R132H or wild-type.
- For peptide-coated ELISA analyzing IDH1R132H-specific IgG in serum, the cut-off for the optical density related to MOG control was defined to be 5.

3.2. Secondary Endpoints

The secondary endpoints are:

- the IDH1R132H-specific T-cell response as number of T-cells detected by IFN-γ ELIspot at visits 3, 5, 7, 10, 12, and 13,
- the IDH1R132H-specific antibody response as titer detected by peptide-coated ELISA at visits 3, 5, 7, 10, 12, and 13, .
- ORR, defined as the proportion of patients showing complete response (CR), partial response (PR) or stable disease (SD) at EOS compared to the baseline value (MRI at visit 2 for ORR under trial drug; MRI prior to any genotoxic therapy (radio-/,

chemotherapy) for ORR of the complete therapy regime). ORR analysis will be based on the central disease assessment according to the RANO criteria (refer to section 7.5.1 of the study protocol),

- PFS, defined as time from the day of first diagnosis to the day of local tumor progression or the day of death of any cause (whichever occurs first), censored by the end of the observation. PFS analysis will be based on the central disease assessment. Patients lacking an evaluation of tumor response (based on radiological or clinical assessment) will have their PFS time censored on the date of first diagnosis with duration of 1 day.

  The end of observation is defined as the date of study termination as indicated by the corresponding entry of the CRF. If this date is not documented, the end of observation is defined as the last documented date.

- Association between immunogenic response (IR) and the clinical outcome parameters (ORR, PFS).

3.3. Further Safety and Tolerability Endpoints

- Adverse Events (AEs):

  Type, intensity (graded by the National Cancer Institute, Common Toxicity Criteria for Adverse Events [CTCAE] V4.0), action taken, outcome, seriousness and relatedness of adverse events (ref. definitions at section 9 of the study protocol).

- Time of onset of AE, defined as the time to the first occurrence of an AE. Patients without AEs will be considered with the last day of treatment or the last observation date (whichever is earlier) as the censoring time.
- Safety laboratory parameters ((hematology, clinical chemistry and urinanalysis, Tumor Marker)
- Electrocardiogram (ECG)
- Physical examination, vital signs, height, weight
- Karnofsky Performane Score (KPS)
- Mini Mental Status Examination (MMSE)
- Testing for HIV, HBV/HCV and tuberculis
- Monitoring of autoimmunity and activation of the immune system
- Molecular screening
- Demographics and other baseline disease characteristics
- Medical history and concomitant diseases
- Previous and concomitant medication

3.4. Covariates

Except for sub-group analysis (ref. section 2.3) further covariates may be used in the statistical analysis, i.e center, gender, age if appropriate.

4. STATISTICAL METHODOLOGY

4.1. General Methodology

The biometrics and data management group at NCT trial center will perform quality control and validation of programming and statistical analysis. SAS current version (i.e. 9.4 or higher) will be used to conduct the analysis.

Concomitant medications will be coded according to the ATC drug dictionary and adverse events according to MedDRA version 20.1.

4.2. Statistical Analysis

4.2.1. Analysis of the Primary Endpoints

The primary analysis consists of summary statistics and interval estimation. For the primary endpoints RLT and IR, summary tables will present the number of patients observed with RLT / with IR, the corresponding percentages and exact 95% CIs according to Clopper-Pearson. These analyses will not be stratified by covariates or adjusted for covariate effects and all conclusions should be based on these primary analyses.

In addition, an exploratory analysis (ref. Section 4.2.2) using subpopulations (ref. section 2) will be carried out.

4.2.2. Analysis of the Secondary and exploratory endpoints

All secondary variables will be analyzed using explorative and mainly descriptive methods:

There will be counting of the absolute and relative frequencies (percentages) for categorical variables. Percentages will include all non-missing values (=100%). Discrete variables will be summarized descriptively for each visit as categorical parameters. Two-sided exact 95% confidence intervals will be constructed as appropriate. When indicated, standard chi-squared tests will be performed to compare frequencies in subgroups. In case of rare events the Fisher's exact test will be used.

- Continuous variables and changes (differences/ or fold changes=ratio of final value to initial value) from the baseline assessment will be summarized descriptively for each visit as numerical parameters using standard measures of central tendency and dispersion. This will include number of observations (n, nmiss), mean, standard deviation, minimum, maximum, and median. When indicated, a paired t-test or Wilcoxon signed-rank test will be performed to compare changes from baseline. All statistical tests will be two-tailed to a significance level of 5%.
- Summary statistics and interval estimation of the endpoints RLT and IR, by treatment group (ref. 2.3) and total. Summary tables will present the number of patients observed with RLT / with IR, the corresponding percentages and exact 95% CIs according to Clopper-Pearson.
- The number of patients with the IDH1R132H-specific antibody response as titer detected by peptide-coated ELISA at the respective study visits will be tabulated by degree of dilution and presented graphically using scatterplots overlaid on boxplots (proc sgplot).

- The ORR proportions by treatment group and total will be given along with the corresponding percentages and exact 95% CIs according to Clopper-Pearson-.
- The survival function for PFS will be estimated using the Kaplan-Meier method implemented in SAS (proc lifetest), including estimations for the median PFS with 95% CI as provided by proc lifetest.
- To examine the association between the immunogenicity data and the clinical response (ORR) as dichotomous variable a logistic regression will be performed. Furthermore, the association between the immunogenicity data and the PFS will be analyzed by cox proportional hazards regression.

4.2.3. Safety Data Analysis

This analysis includes summaries of the following categories of safety data for the safety set:

- Adverse Events (AEs and SAEs, AEs leading to withdrawal, common AEs)

  Adverse events will be coded according to MedDRA 20.1. AEs will be grouped by body system and the preferred term will be used.

  The grading of all AEs listed in the CTCAE v4.0 will be based on the information contained therein.

  The grading of all other AEs, i.e. those which are not listed in the CTCAE v4.0, will be based on the following definitions:

**mild:**	temporary event which is tolerated well by the patient.
**moderate:**	event which results in discomfort for the patient and impairs his/ her normal activity.
**severe:**	event which results in substantial impairment of normal activities of patient.

  Summary tables with the number of patients observed with AEs (all causalities and drug related) and corresponding percentages will be presented.

  In Addition the most common AEs (all causalities and drug related) will be reported. Most common AEs: those AEs occurring in at least 10% of the treatment).

  Furthermore a listing by patient will be presented including the following information for each AE: a description of the event, start date /end date, seriousness, severity, action taken, causal relationship with the Peptid/ Imiquimod/TMZ, and outcome.

  The analysis of the time to the first occurrence of an (first) AE (weeks since start of vaccination) will be carried out with the Kaplan-Meier estimator. The hazard rate, indicating the risk to experience a (first) AE over time, will be estimated with the life table method for selected time intervals.

  An association between the appearance of AE and the vaccination will be analyzed by indicating the absolute and relative frequencies for all and for drug related AEs by number of vaccines.

- Clinical Laboratory Investigations

Summary tables will be prepared to examine the distribution of laboratory measures over time. The number of patients with laboratory values that are below, within, or above normal ranges will be tabulated for each parameter. Descriptive summaries (mean, SD, median, minimum, and maximum) of actual values and of changes from baseline will be presented for each parameter. Boxplots will be prepared, if required.

- Hemodynamics and performance status

  Descriptive summaries of actual values and changes from screening/baseline to last observation will be calculated for vital signs (SBP, DBP, and HR), body weight, temperature, Karnofsky Performance Status and Mini Mental Status Examination (MMSE)

- ECG Investigations

  The number and percentage of patients with normal sinus rhythm and normal and abnormal ECG results at baseline and follow-up will be tabulated.

4.2.4. Further Analyses

Patient disposition (the number of patients enrolled, treated, and in the analysis populations) will be tabulated. In addition, the number of patients who withdrew from the study and reasons for discontinuation will be summarized. Physical examinations, prior and concomitant medications, and vital signs will be recorded by visit. Study drug administration and reasons for the deviations from the planned therapy will be tabulated.

4.3. Statistical/Analytical Issues

4.3.1. Adjustments for Covariates

No covariates will be used in the primary efficacy analysis. All other statistical tests are of an exploratory nature. No adjustment for covariates will be done.

4.3.2. Multiple Comparisons/Multiplicity

No multiple testing procedures will be used in the primary efficacy analysis. All other statistical tests are of an exploratory nature. No adjustment for multiplicity will be done.

4.3.3. Handling of Dropouts, Missing Data, and Outliers

Missing values will not be replaced or imputed. For patients with incomplete follow-up, time to last follow-up date will be used as the censoring time in the analysis of time-to-event data.

Data of patients withdrawing from the study will be used only up until the date of the withdrawal, unless their consent to use further data (e.g. from clinical follow-up) is expressly given and documented in their medical records.

Imputation rules for partial or missing dates in the AE/ or the Concomitant Medication data are given in SAP_Appendix A3.

4.3.4. Center Pooling Method and Handling Center Effects

The primary analysis will not be stratified by center or adjusted for center effects. However, for descriptive purposes additional results will be presented for each center separately if apropriate.

4.3.5. Interim Analyses and Data Monitoring

In this trial early stopping rules have been implemented to detect unacceptable rates of toxicities (frequency of RLTs) or unexpected shortening of PFS, ref. also section 1.7.

5. STATISTICAL REPORTING

5.1. Study Patients and Data Sets Analyzed

The frequency and percent of patients in each population, study withdrawals, subgroups, and major protocol violations will be presented.

Flow of patients through the study will be displayed in a "CONSORT" diagram http://www.consort-statement.org/consort-statement/flow-diagram0/, with number of patients screened, included and analysed. Reasons for exclusion of non-included patients, lost to follow up/ discontinuations will be displayed.

5.2. Demographic and Patient Characteristics

Descriptive summaries in the safety set at screening, baseline and follow up visits (as indicated) will be presented for

- Demographics and other baseline characteristics as tumor diagnosis
- Medical history
- Molecular screening and molecular Assessment
- Pregnancy Test
- Previous anticancer treatments
- Prior and concomitant medication
- Karnofsky Performance Status
- Mini Mental Status Examination (MMSE)
- HIV/HBV/HCV-Serology (negative /positive) at screening
- Tuberculosis test (positive /negative) at screening

5.3. Evaluation of Treatment Compliance and Exposure

The following items will be described in the safety set:

- Number of patients with Corticosteroid Medication –previous and concomitant - Total and by Drug name
- Number of patients with previous and concomitant medication other than Corticosteroid Medication - overall and broken down by Drug
- Number of patients with Radiotherapy - overall and broken down by localization/radiation site
- Duration of Radiotherapy (Last radiation date – first radiation date)
- Number of fractions per patient (mean, median, minimum, maximum)
- Number of patients with concomitant Temozolomid Therapy
  Number of patients with treatment interruptions /dose modifications
- Duration of Treatment (days/months)
- Duration of Observation (days/months)
- Evaluability Status

- Patient Status at EOT/EOS

The following items will be analyzed in the safety set for the Concomitant Temozolomid-Monotherapy and radiation therapy:

- Number of treatment cycles per patient (mean, median, minimum, maximum)
- Number of patients having 1,2,3,... treatment cycles (frequency distribution)
- Number of patients with dose modifications
- Number of patients definitely withdrawing from treatment
- Number of patients completing the treatment as planned (i.e., without any dose reductions)
- Summaries (mean, median, minimum, maximum) of cumulative dose of drugs and radiation (if applicable)
- Number of patients receiving concomitant medications, overall as well as broken down by type of medication.

5.4. Immunogenicity Parameters

5.4.1. Primary immunogenicity endpoint immunogenic response

The number and percentages of patients with IR with two-sided 95% confidence intervals (CI) according to Clopper-Pearson- will be presented.

5.4.2. Autoimmunity

The following items will be described in the immunogenicity set:

- Autoimmuniity laboratory parameters at screening and follow-up
- Immune monitoring at screening and follow-up

5.4.3. (Reccurrent) Tumor Tissue

The following items will be described in the safety set for patients suffering from tumor recurrence:

- Surgery done (yes/No)
- Biopsy done (yes/no)
- Analysis of IDH1R123H Mutation Status performed: (local/ central)
- IDH1-mutations (Present/Absent)
- Fresh tissue of the recurrent tumor collected (yes/no)

5.5. IDH1 Peptide Vaccination/ Imiquimod/Aldara

The following items will be described in the safety set by study visit:

- Site of s.c. vaccination
- Total dose of 300 µg peptide in 1.4 ml Montanide injected (yes/no)

- Time difference between IDH1 peptide vaccination and imiqumod/aldara administration
- Adverse reactions/ complications occured after the administration of vaccine (yes/no)
- Deviations from scheduled application

5.6. Analysis of the Primary Safety Endpoint

5.6.1. Regime-Limiting Toxicity (RLT)

Descriptive summaries in safety set will be presented:

- Type of RLT and relationship to IMP
- Number and percentages of patients with at least one RLT with two-sided 95% confidence intervals (CI)
- Number of patients with at least one RLT per CTCAE severity grade and total
- All toxicity notifications (listing by patient)

5.7. Analysis of Further Safety Endpoints

5.7.1. Adverse Events (AEs)

AEs will be grouped by body system and the preferred term should be used. CI will be calculated for the percentage of patients with AEs according to Pearson-Clopper.

The following items will be described in the safety set.

- AEs (all causalities/ treatment related)
- The incidence of AEs.
- The common AEs (e.g. those in at least 10% of the treatment) with two-sided 95% confidence intervals (CI)
- Number of patients with at least one AE per CTCAE severity grade
- All SAE (all causalities and treatment related)
- Number and percentages of patients with at least one SAE with two-sided 95% confidence intervals (CI)
- All SAE notifications data (listing by patient)

5.7.2. Clinical Laboratory Evaluation

Each clinical laboratory variable will be described in the safety set by study visit.

5.7.3. Vital Signs, Physical Findings and Other Observations Related to Safety

The following items will be described in the safety set.

- Vital signs and physical examination
- 12-lead Electrocardiogram

5.8. Analysis of the Clinical Outcomes

Descriptive summaries in the safety set will be presented:

- The ORR and with two-sided 95% confidence bounds

- Tumor assessments according to the local and central RANO criteria at baseline and follow-up visits. The tumor assessment will be described by giving absolute numbers and percentages for each response category (CR/PR/SD/PD).
- Summaries of cumulative event rates (PFS,OS)

6. REPORTING CONVENTIONS ANDPLANNED DATA DISPLAYS

The proposed reporting conventions, and the content and formatting of tables, listings, and figures are displayed in the SAP Appendix A2.

7. REFERENCES

- ICH Guidelines E3 “Structure and Content of Clinical Study Reports” http://www.ich.org/products/guidelines/efficacy/article/efficacy-guidelines.html
- ICH Guidelines E9 “Statistical Principles for Clinical Trials” http://www.ich.org/products/guidelines/efficacy/article/efficacy-guidelines.html
- D. Moher, K. F. Schulz, D. G. Altman (2010) The CONSORT statement: revised recommendations for improving the quality of reports of parallel group randomized trials. BMC Med Res Methodol, 1(1).

8. APPENDICES

A1: SAP Appendix A1_Protocol Deviations

A2: SAP Appendix A2_TFL

A3: SAP_Appendix A3: Imputation rules for partial or missing dates
